# The Human Nuclear Poly(A)-Binding Protein Promotes RNA Hyperadenylation and Decay

**DOI:** 10.1371/journal.pgen.1003893

**Published:** 2013-10-17

**Authors:** Stefan M. Bresson, Nicholas K. Conrad

**Affiliations:** Department of Microbiology, University of Texas Southwestern Medical Center, Dallas, Texas, United States of America; Stanford University School of Medicine, United States of America

## Abstract

Control of nuclear RNA stability is essential for proper gene expression, but the mechanisms governing RNA degradation in mammalian nuclei are poorly defined. In this study, we uncover a mammalian RNA decay pathway that depends on the nuclear poly(A)-binding protein (PABPN1), the poly(A) polymerases (PAPs), PAPα and PAPγ, and the exosome subunits RRP6 and DIS3. Using a targeted knockdown approach and nuclear RNA reporters, we show that PABPN1 and PAPα, redundantly with PAPγ, generate hyperadenylated decay substrates that are recognized by the exosome and degraded. Poly(A) tail extension appears to be necessary for decay, as cordycepin treatment or point mutations in the PAP-stimulating domain of PABPN1 leads to the accumulation of stable transcripts with shorter poly(A) tails than controls. Mechanistically, these data suggest that PABPN1-dependent promotion of PAP activity can stimulate nuclear RNA decay. Importantly, efficiently exported RNAs are unaffected by this decay pathway, supporting an mRNA quality control function for this pathway. Finally, analyses of both bulk poly(A) tails and specific endogenous transcripts reveals that a subset of nuclear RNAs are hyperadenylated in a PABPN1-dependent fashion, and this hyperadenylation can be either uncoupled or coupled with decay. Our results highlight a complex relationship between PABPN1, PAPα/γ, and nuclear RNA decay, and we suggest that these activities may play broader roles in the regulation of human gene expression.

## Author Summary

In eukaryotes, mRNAs include a stretch of adenosine nucleotides at their 3′ end termed the poly(A) tail. In the cytoplasm, the poly(A) tail stimulates translation of the mRNA into protein, and protects the transcript from degradation. Evidence suggests that poly(A) tails may play distinct roles in RNA metabolism in the nucleus, but little is known about these functions and mechanisms. We show here that poly(A) tails can stimulate transcript decay in the nucleus, a function mediated by the ubiquitous nuclear poly(A) binding protein PABPN1. We find that PABPN1 is required for the degradation of a viral nuclear noncoding RNA as well as an inefficiently exported human mRNA. Importantly, the targeting of RNAs to this decay pathway requires the PABPN1 and poly(A) polymerase-dependent extension of the poly(A) tail. Nuclear transcripts with longer poly(A) tails are then selectively degraded by components of the nuclear exosome. These studies elucidate mechanisms that mammalian cells use to ensure proper mRNA “quality control” and may be important to regulate the expression of nuclear noncoding RNAs. Furthermore, our results suggest that the poly(A) tail has diverse and context-specific roles in gene expression.

## Introduction

Prior to their export to the cytoplasm, nuclear pre-mRNAs must be capped, spliced, polyadenylated, and assembled into export-competent messenger ribonucleoprotein particles (mRNPs). Mistakes in any of these processes lead to aberrant mRNAs that may code for proteins with deleterious effects. As a result, cells have developed RNA surveillance or quality control (QC) mechanisms that preferentially degrade misprocessed transcripts [1]–[3]. While the mechanisms and factors involved in nuclear RNA quality control have been extensively studied in yeast models, these pathways remain largely uncharacterized in metazoans.

The addition of a poly(A) tail is essential for normal mRNA biogenesis, but polyadenylation can stimulate RNA QC pathways in *S. cerevisiae*. The yeast Trf4-Air2-Mtr4 polyadenylation (TRAMP) complex is a co-factor for the degradation of aberrant rRNA, tRNA, snoRNAs, snRNAs, long noncoding RNAs (lncRNAs), and mRNAs [2], [4]–[7]. Decay is carried out by the nuclear exosome, a nine-subunit complex that associates with the Rrp6 and Dis3 nucleases [5], [8]. In addition to polyadenylation by TRAMP, a noncanonical poly(A) polymerase not involved in 3′end formation of mRNAs, predicted RNA QC targets can be hyperadenylated by Pap1, the canonical poly(A) polymerase (PAP) that polyadenylates mRNA 3′-ends [9]–[13], but whether this is directly linked to decay is unclear. Although the mechanisms linking polyadenylation with decay remain unknown, the nuclear poly(A)-binding protein (PABP), Nab2, recruits the exosome to polyadenylated RNAs, suggesting that yeast PABPs couple hyperadenylation with decay [14].

Few studies on mammalian nuclear RNA QC systems have been published, but some roles of the poly(A) tail in *S. cerevisiae* appear to be conserved in mammals. For example, mammalian TRAMP homologs promote polyadenylation and decay of aberrant rRNA and unstable promoter-associated transcripts [15]–[17]. Furthermore, polyadenylation induced by a Kaposi's sarcoma-associated herpesvirus (KSHV) host shut-off protein results in the hyperadenylation and destabilization of host transcripts [18]. Both yeast and mammalian mRNAs are hyperadenylated upon inhibition of bulk mRNA export [13], [19], [20]. In addition, knockdown of exosome components leads to the accumulation of oligoadenyated nuclear RNAs [21]. Thus, certain aspects of poly(A) tail functions in nuclear RNA QC appear to be conserved in mammals, but little empirical evidence has been reported and mechanistic details remain largely unknown.

Our previous studies using the noncoding KSHV polyadenylated nuclear PAN RNA further support the idea that the poly(A) tail plays an important role in mammalian nuclear RNA decay. PAN RNA is a polyadenylated, capped, RNA polymerase II (pol II) transcript that accumulates to high levels in the nucleus, thereby making it a useful model to study nuclear RNA decay pathways. The high nuclear levels of PAN RNA depend on the presence of a 79-nt stability element near its 3′ end termed the ENE [22]–[24]. The ENE interacts with the poly(A) tail in cis, protecting the transcript from degradation [23]–[25]. In addition, the ENE stabilizes an intronless β-globin reporter mRNA leading to its accumulation in the nucleus. Due to the coupling of mRNA export with splicing, this intronless mRNA is less efficiently exported than its spliced counterparts [26], [27] and therefore likely succumbs to the RNA QC machinery. These observations led to the model that inefficiently processed or exported transcripts are rapidly degraded in the nucleus, and implied that the presence of a poly(A) tail is linked to their degradation. Importantly, the factors involved in this decay pathway were not previously described.

Comparison of nuclear PABP functions in mRNA 3′-end formation and nuclear RNA decay suggest important mechanistic distinctions among fission yeast, budding yeast, and mammals. The human nuclear PABP, PABPN1, stimulates PAP processivity and controls poly(A) length in 3′ processing reactions in vitro [28]. Recent studies suggest that PABPN1 also regulates alternative polyadenylation of specific mRNAs [29], [30]. The *S. pombe* nuclear PABP, Pab2, is homologous to PABPN1, but is not related to *S. cerevisiae* Nab2. Pab2 promotes the exosome-mediated nuclear RNA decay of specific pre-snoRNAs, pre-mRNAs, and meiotic mRNAs [31]–[34]. However, *S. pombe* Pab2 does not stimulate PAP activity [28] and deletion mutants in Pab2 are viable [35], demonstrating that it is not essential for the polyadenylation of most mRNAs. Moreover, Pab2 or Nab2 depletion causes bulk poly(A) tail hyperadenylation [35], [36], whereas depletion of PABPN1 homologs in mouse and *Drosophila* decrease steady-state poly(A) tail lengths [37], [38]. These important distinctions in PABP function highlight the difficulties in extrapolating from one system to another, and underscore the need for detailed mechanistic studies in higher eukaryotes.

Here we define a pathway that promotes the degradation of polyadenylated nuclear RNAs in mammalian cells. This pathway depends on PABPN1, the canonical PAPs, PAPα and PAPγ, and the nuclear exosome components RRP6 and DIS3. The pathway targets ENE-lacking PAN RNA and intronless β-globin mRNA reporters, but does not degrade a spliced β-globin mRNA. Interestingly, PANΔENE RNA and intronless β-globin RNAs are polyadenylated upon PABPN1 knockdown or PAPα and PAPγ co-depletion, but the poly(A) tails are substantially shorter. In contrast, co-depletion of RRP6 and DIS3 stabilizes hyperadenylated forms of both intronless β-globin mRNA and PANΔENE RNA reporters and this hyperadenylation depends on PABPN1 and canonical PAP activity. Bulk RNA analysis reveals that a significant fraction of newly synthesized cellular polyadenylated RNAs is subject to PABPN1-dependent hyperadenylation. Efficiently spliced endogenous mRNAs appear to be unaffected by this decay pathway, but an endogenous polyadenylated nuclear noncoding RNA is, consistent with the interpretation that this pathway targets non-exported RNAs. Together, our results suggest that PABPN1 and the PAPs, PAPα and PAPγ, promote the exosome-mediated degradation of nuclear polyadenylated transcripts in human cells.

## Results

### PABPN1 is required for rapid PANΔENE RNA decay

**Figure 1:**
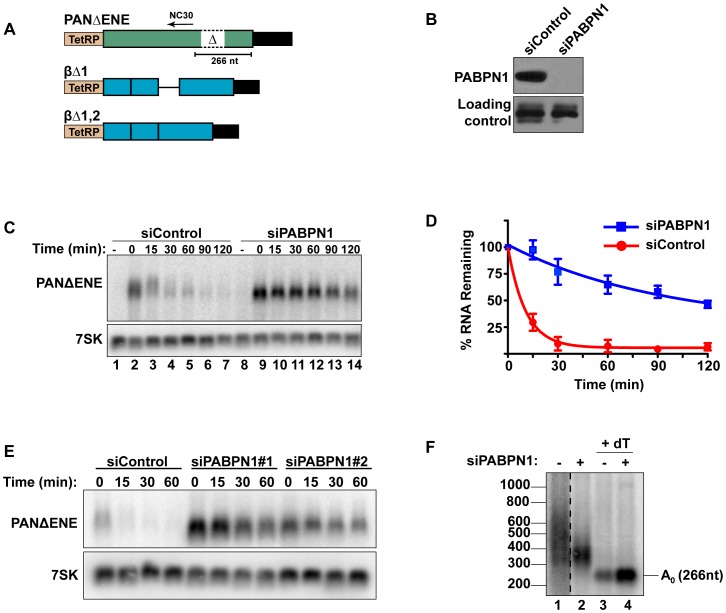
Rapid degradation of PANΔENE requires PABPN1. (**A**) Diagram of the TetRP-driven PANΔENE and β-globin reporters. PANΔENE has a deletion of the 79-nt ENE [23]. The position of oligonucleotide NC30 is shown (arrow). The β-globin reporters lacked either the first (βΔ1), or both (βΔ1,2), introns. (**B**) Western blot analysis of extracts from cells transfected with either a non-targeting control siRNA or a two-siRNA pool targeting PABPN1. Protein extract was probed with antibodies against either PABPN1 or PABPC1 (loading control). (**C**) Representative transcription pulse assay; the “-” lane samples were harvested prior to the two-hour transcription pulse. 7SK serves as a loading control (bottom). (**D**) Decay curves of the transcription pulse assay data (*n = 3*). The data were fit to two-component exponential decay curves as described in the Materials and Methods. (**E**) Northern blot analysis of a transcription pulse-chase assay in cells transfected with a nontargeting control siRNA or either of two independent siRNAs against PABPN1. (**F**) Poly(A) tail length analysis of PANΔENE as described in text. Lane 1 is displayed at a darker exposure as indicated by dotted vertical lines.

To measure PANΔENE RNA stability, we used a well-characterized transcription pulse-chase assay [24], [39], [40]. In this assay, a plasmid that expresses PANΔENE under control of a tetracycline-responsive promoter (TetRP)(Figure 1A) is transfected into cells stably expressing a tetracycline-responsive transcriptional activator in the presence of doxycycline (dox) to repress transcription. Transcription is induced for 2 hr by dox removal, then repressed by the readdition of dox, cells are collected over time and PANΔENE RNA levels are followed by northern blot. We previously demonstrated that PANΔENE RNA decay follows two-component decay kinetics [24]. A subpopulation of transcripts is degraded rapidly (defined herein as t_1/2_≤15 min), while the remaining RNA pool is degraded more slowly. Regression analysis allows us to estimate the percentage of transcripts in each pathway and their corresponding half-lives. The presence of the ENE protects polyadenylated RNAs from the rapid decay pathway, but the cellular factors driving rapid decay have not been previously elucidated.

Interactions between the ENE and the poly(A) tail stabilize PAN RNA, suggesting that a poly(A) tail may promote rapid RNA decay. Because PABPN1 binds to poly(A) tails in the nucleus and the *S. pombe* PABPN1 homolog promotes RNA decay, we tested the effects of PABPN1 depletion on rapid PANΔENE decay. We used siRNAs to efficiently (∼95%) knockdown PABPN1 in 293A-TOA cells [39] (Figure 1B, Figure S1A and S1B). Transcription pulse-chase assays demonstrated that RNA decay was impaired upon PABPN1 depletion (Figure 1C). Two different PABPN1 siRNAs yielded similar results, consistent with a PABPN1-specific effect (Figure 1E, Figure S1C). Fitting the data to two-component exponential decay curves (Figure 1D) showed that nearly all of the RNA degraded rapidly in the control cells (∼95%, t_1/2_∼7 min, S1), but predicted that only ∼6.6% of the transcripts had t_1/2_≤15 min in PABPN1-depleted cells. Thus, we conclude that PABPN1 is necessary for the rapid decay of PANΔENE RNA in vivo.

Because hyperadenylation often correlates with decay and PABPN1 stimulates PAP activity, we were interested in the poly(A) tail lengths of PANΔENE RNA in the presence or absence of PABPN1. RNA samples taken immediately after the 2-hr transcription pulse (T = 0) were cleaved with RNase H targeted by DNA oligonucleotide complementary to a region near the PANΔENE RNA 3′ end (NC30, Figure 1A), and the 3′ fragment was detected by northern blot using a 3′-end specific probe. A very broad smear was observed in the control cells (Figure 1F, lane 1) and this smear collapsed into a single discrete product upon addition of oligo dT_40_ to the RNase H reaction (Figure 1F, lane 3) demonstrating that the smear was due to heterogeneous poly(A) tail lengths. We estimated that the poly(A) tail distribution in the control cells corresponds to ∼50–400 adenosines. Notably, this broad distribution of transcripts was lost when PABPN1 was depleted (compare lanes 1 and 2), but the transcripts maintained a sizable poly(A) tail of ∼50–150 nt. Taken together, these data suggest that PABPN1 is necessary for the hyperadenylation and rapid decay of PANΔENE RNA.

### PABPN1 is required for the rapid degradation of an intronless β-globin mRNA

**Figure 2:**
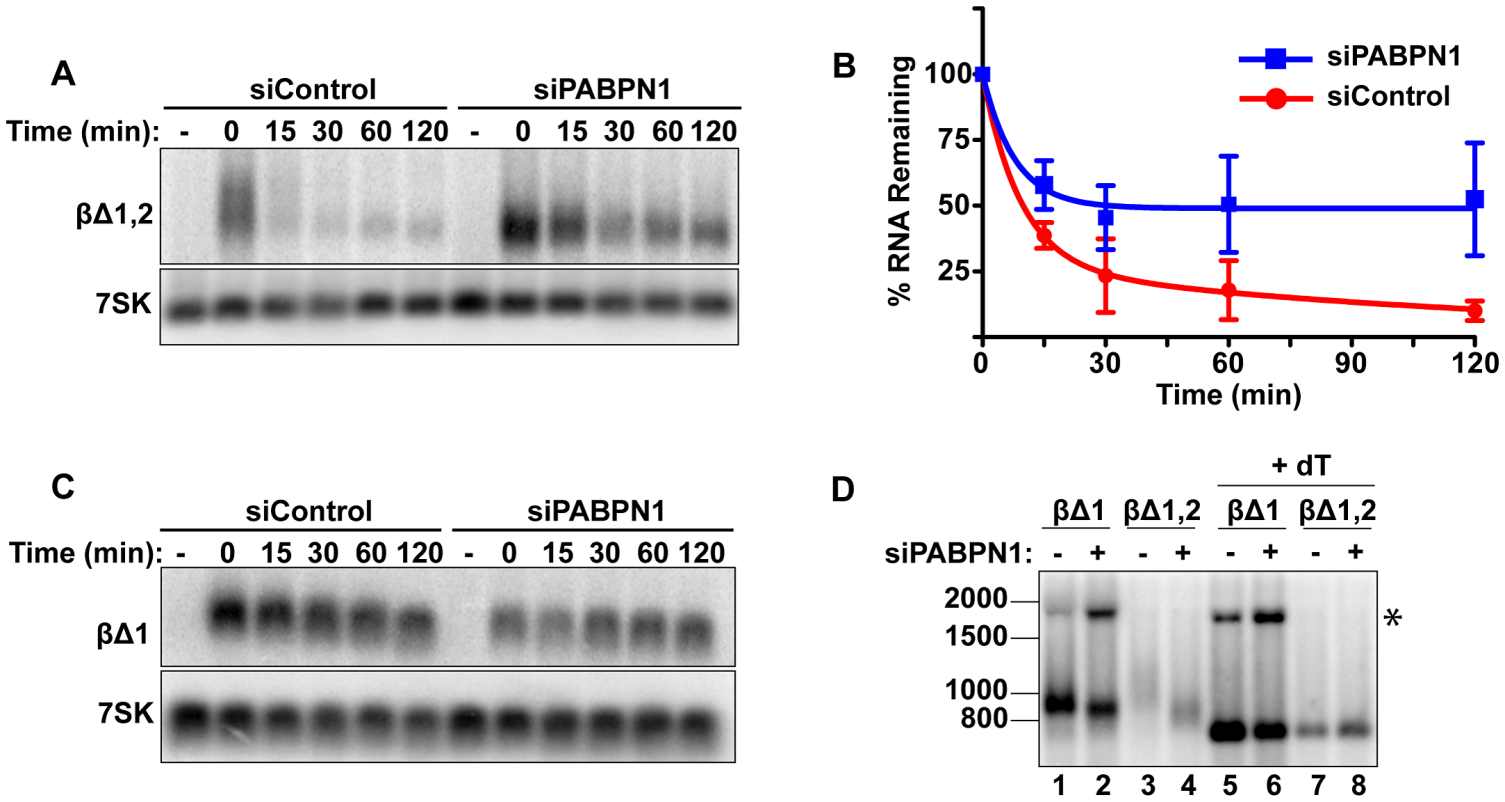
Rapid degradation of intronless β-globin requires PABPN1. (**A**) Representative transcription pulse assays using the intronless reporter (βΔ1,2) and cells transfected with the indicated siRNAs. (**B**) Decay curves of biological replicates of the transcription pulse assay data (*n = 3*). (**C**) Representative transcription pulse assay of the spliced reporter (βΔ1). (**D**) Poly(A) tail analysis of βΔ1 and βΔ1,2. Time zero samples from cells transfected with the specified siRNAs was treated with RNase H and oligo(dT) as indicated. The β-globin pre-mRNA is denoted by an asterisk (*).

To determine whether human mRNAs are subject to PABPN1-dependent decay, we initially examined the effects of PABPN1 knockdown on two TetRP-driven β-globin reporter constructs. One contains no introns (βΔ1,2) while the other retains the second intron of β-globin (βΔ1)(Figure 1A). Because pre-mRNA splicing promotes nuclear export, the inefficiently exported βΔ1,2 mRNA is unstable, presumably due to nuclear RNA QC [24], [27], [41]. However, a fraction of the intronless β-globin is exported and this fraction is subject to slower decay in the cytoplasm [24], [42]. Similar to PANΔENE, PABPN1 knockdown stabilized the intronless β-globin transcript (Figure 2A, 2B). Furthermore, the decay kinetics were consistent with two component exponential decay. In this case, ∼73% of the βΔ1,2 was degraded rapidly in the control cells, but only ∼51% was subject to rapid decay upon PABPN1 knockdown (Table S1). In contrast, the spliced β-globin reporter mRNA was largely unaffected by PABPN1 knockdown and was stable over two-hour (Figure 2C) or eight-hour time courses (Figure S2). Thus, β-globin mRNA generated from an intronless gene is subject to PABPN1-dependent degradation, but the same mRNA produced from a spliced intron-containing gene is not.

We further examined the effects of PABPN1 knockdown on the poly(A) tail lengths of the intronless and spliced β-globin RNAs after a two-hour transcription pulse (Figure 2D). The intronless β-globin poly(A) tails closely resembled those of PANΔENE. That is, in the control cells, the poly(A) tails were longer and more heterogeneous than in the PABPN1 depleted cells (lanes 3 and 4). In contrast, the spliced β-globin mRNAs were less heterogeneous in either the presence or absence of PABPN1 and the difference in mobility between samples was smaller (lanes 1 and 2). Interestingly, the size of the intronless β-globin poly(A) tails in the absence of PABPN1 were more similar to those of the spliced β-globin RNA, consistent with the idea that the longer poly(A) tails were due to transcript hyperadenylation in control cells and not to hypoadenylation in the absence of PABPN1. Additionally, poly(A) tail analysis of the stable wild type version of PAN RNA revealed that it had a shorter poly(A) tail than PANΔENE, its unstable counterpart (data not shown). Taken together, these data suggest that unstable nuclear transcripts have longer poly(A) tails, and that PABPN1 is required for this poly(A) tail extension. For the purposes of this paper, we will describe the transcripts with PABPN1-dependent long poly(A) tails as “hyperadenylated”. For PANΔENE, hyperadenylated transcripts have a poly(A) tail of roughly 150–400 nucleotides.

### Polyadenylation by PAPα/γ promotes PANΔENE RNA decay

**Figure 3:**
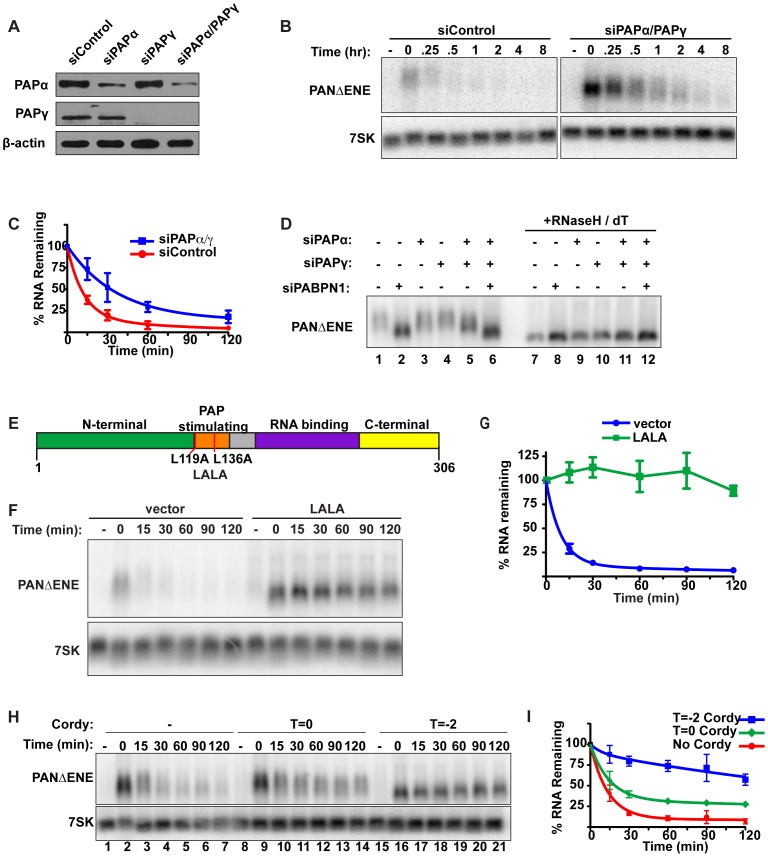
Polyadenylation is required for PANΔENE RNA decay. (**A**) Western blot analysis of cells transfected with either a control siRNA or siRNA pools targeting PAPα, PAPγ, or both. Specific proteins were detected using antibodies against PAPα, PAPγ, or β-actin (loading control). (**B** and **C**) Representative transcription pulse assay and decay curves of PANΔENE from cells transfected with the indicated siRNAs *(n≥4)*. (**D**) Length analysis of PANΔENE from cells transfected with the indicated siRNAs. RNA was harvested following a two-hour transcription pulse (T = 0). The samples in lanes 7–12 were treated with RNase H and oligo(dT) prior to northern blot analysis. (**E**) Schematic showing the domain structure of PABPN1 as well as the mutations made to generate the dominant negative construct, LALA. (**F**) Results from a transcription pulse from cells transfected with the dominant negative allele LALA. (**G**) Decay curves from a transcription pulse as shown in (**F**)*(n≥3)*. (**H** and **I**) Representative transcription pulse assay and decay curves of PANΔENE performed in the presence or absence of cordycepin *(n = 3)*. Cordycepin was added either during (T = −2) or after (T = 0) the transcriptional pulse.

PABPN1 stimulates processive polyadenylation by PAPα (also known as PAP II, PAPOLA) [28] and stabilization of PANΔENE and βΔ1,2 by PABPN1 knockdown correlates with shorter poly(A) tails. Therefore, we reasoned that extension of poly(A) tails by PAPα may be linked to rapid PANΔENE decay. To assess the requirement of PAPα, and its close homolog PAPγ (neo-PAP, PAPOLG) [43], [44], for PANΔENE hyperadenylation and decay, we monitored PANΔENE stability upon siRNA-mediated knockdown of PAPα and PAPγ. We achieved robust knockdown of PAPγ, and substantial, though incomplete, knockdown of PAPα (Figure 3A). PANΔENE RNA was stabilized when both enzymes were depleted (Figure 3B, 3C), but not upon knockdown of either PAP individually (Figure S3A), suggesting functional redundancy between PAPα and PAPγ. In addition, different combinations of PAPα and PAPγ siRNAs also stabilized PANΔENE RNA, supporting the conclusion that stabilization was not due to off-target effects of siRNAs (Figure S3B). Notably, PAPα and PAPγ knockdown did not stabilize PANΔENE RNA to the same extent as PABPN1 knockdown. We suggest this is due to incomplete knockdown of PAPα compared to the highly efficient knockdown of PABPN1. Additionally, because PAPs are catalytic rather than stoichiometric factors, low protein levels may be sufficient for function. Even so, the protection of PANΔENE RNA from rapid decay upon PAPα and PAPγ co-depletion supports a role for the canonical PAPs in nuclear RNA decay.

We next examined the effects of PAPα and PAPγ depletion on PANΔENE poly(A) tail length (Figure 3D). Consistent with the stability data, knockdown of either PAP individually had little effect on RNA length (compare lanes 1–4), but co-depletion of PAPα and PAPγ reduced the poly(A) tail length of PANΔENE (lane 5). The decrease in poly(A) tail length was less than that observed upon PABPN1 knockdown (compare lanes 2 and 5), mirroring the effects on stability. Importantly, no additive decreases in poly(A) tail length were observed when all three factors were depleted (lane 6), consistent with the proteins functioning in the same pathway. We conclude that PABPN1 and the canonical PAPs work in concert to hyperadenylate the unstable PANΔENE RNA.

The results above show that depletion of either PABPN1 or the canonical PAPs stabilizes transcripts that would otherwise be rapidly degraded. Because PABPN1 directly promotes PAP activity [28], our data suggest that PABPN1-dependent stimulation of hyperadenylation promotes decay of targeted transcripts. If this model is correct, overexpression of a mutant PABPN1 defective in PAP stimulation activity should block decay. To test this prediction, we introduced an L119A, L136A (“LALA”) mutation into PABPN1 (Figure 3E). Previous work has shown that the resulting protein binds poly(A) with similar affinity to wild type protein, but is unable to stimulate polyadenylation by PAPα [45]–[47]. As predicted, PANΔENE RNA had a substantially shorter poly(A) tail upon LALA overexpression, similar to the sizes observed upon PABPN1 knockdown (Figure S3C, S3D). More importantly, the resulting RNA was more stable than the controls (Figure 3F, 3G), suggesting that PABPN1 must be able to stimulate polyadenylation in order to promote decay. Taken together, these results argue that PABPN1 stimulation of polyadenylation by PAP is important for its role in RNA decay.

Several pieces of data suggest that poly(A) tail extension precedes the rapid nuclear RNA decay observed here. First, the polyadenylation factors PABPN1 and the canonical PAPs are required for rapid decay. Second, the PAP-stimulating activity of PABPN1 is necessary for decay. Third, stabilization of PANΔENE or intronless β-globin RNA correlates with shorter poly(A) tails. Fourth, transient increases in PANΔENE length are sometimes observed at the earliest time points after transcription inhibition (e.g. Figure 1C compare lanes 2 and 3). To test this hypothesis with another experimental approach, we examined the effects of the polyadenylation inhibitor cordycepin (3′-deoxyadenosine) on PANΔENE RNA. Addition of cordycepin coincident with the induction of transcription (T = −2) resulted in a robust stabilization of PANΔENE (Figure 3H, compare lanes 1–7 with 15–21, and Figure 3I). Comparison of PANΔENE RNA at time zero verified that cordycepin treatment resulted in shorter poly(A) tails (≤50 nt) than the control (Figure S3E). Because cordycepin acts through a chain termination mechanism, it can inhibit transcription as well as polyadenylation. The accumulation of transcripts after the two-hour pulse (Figure 3H, lane 16) and their short poly(A) tail lengths (Figure S3E) show that cordycepin primarily inhibited polyadenylation under our experimental parameters. Similar analyses demonstrated that intronless β-globin was stabilized by the addition of cordycepin (Figure S3F, S3G), whereas the stability of spliced β-globin was unaffected (Figures S3H, S3I). Thus, the generation of PANΔENE with short, cordycepin-terminated poly(A) tails increases PANΔENE half-life.

We next tested the effects of adding cordycepin simultaneously with transcription shutoff (T = 0; Figure 3H, lanes 8–14, Figure 3I). Under these conditions, only polyadenylation occurring after transcription shut-off will be inhibited. Importantly, cordycepin must be converted to cordycepin triphosphate prior to termination of polyadenylation, so the efficiency of inhibition may be limited by the in vivo kinetics of substrate phosphorylation. Even so, we observed a protection of PANΔENE RNA from rapid decay (Figure 3H and 3I). Regression analysis suggests that only ∼67% of transcripts undergo rapid decay when cordycepin is added at T = 0 compared to ∼90% in untreated samples (Table S1). These data suggest that poly(A) tail extension (or re-adenylation), and not strictly length, may be important for rapid RNA decay in vivo (see Discussion). However, we acknowledge that a terminal 3′-deoxyadenosine could inhibit decay factors requiring a 3′ hydroxyl group or the presence of cordycepin in cells may have additional indirect consequences on cellular metabolism (e.g. [48]).

### The nuclear exosome is required for PANΔENE rapid decay

**Figure 4:**
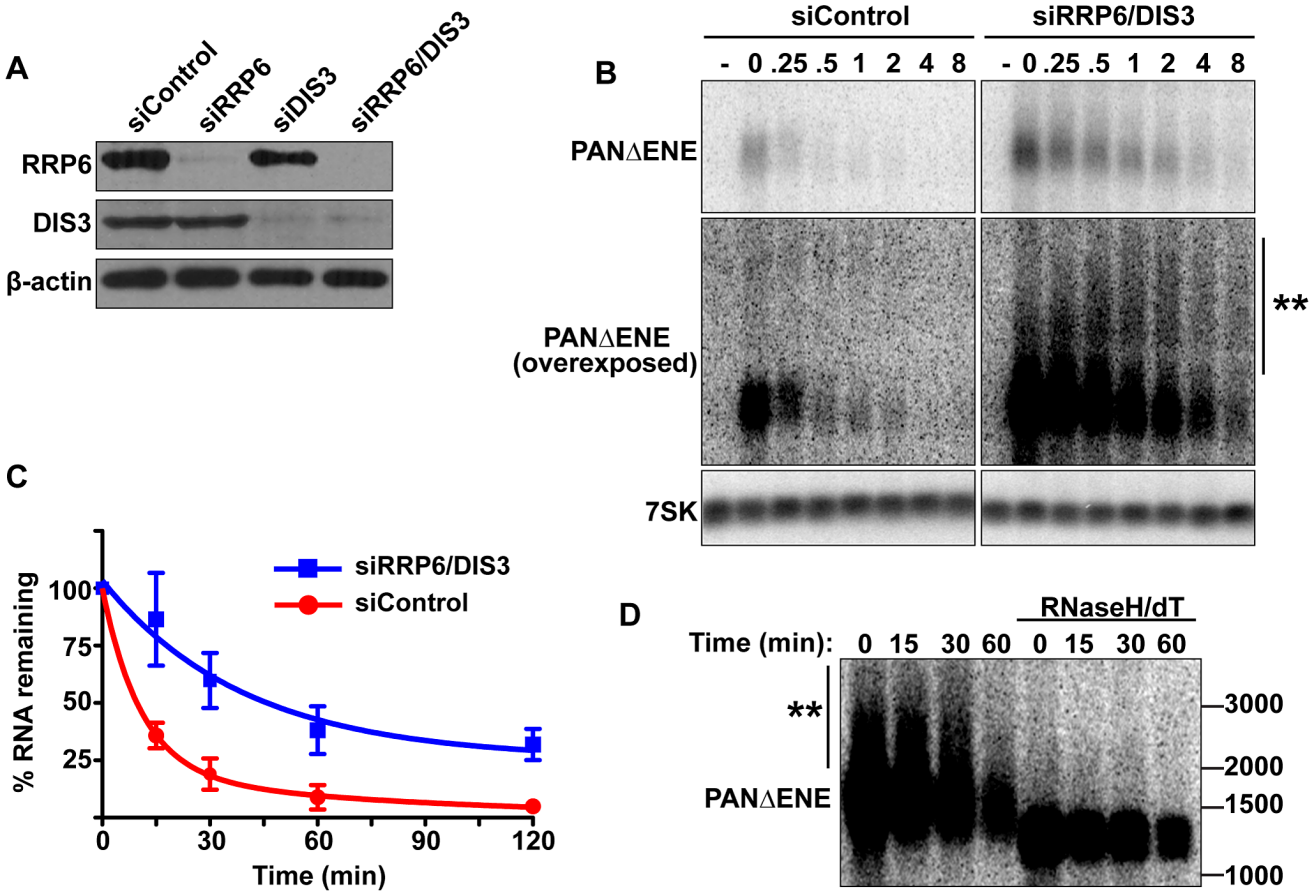
PANΔENE is stabilized and hyperadenylated upon exosome depletion. (**A**) Western blot analysis of cells transfected with either a non-targeting control siRNA or siRNA pools against RRP6, DIS3, or both. Blots were probed with antibodies specific for RRP6, DIS3, and β-actin (loading control). (**B**) *Top*, Representative transcription pulse assay from cells transfected with the indicated siRNAs. The same blot and exposure are shown for siControl and siRRP6/DIS3. *Lower panels*, Overexposed version of the blot and 7SK loading control. (**C**) Decay curves of the transcription pulse assays as shown in (**B**)(*n = 3*). (**D**) Northern blot showing the mobility of PANΔENE RNA from RRP6 and DIS3 depleted cells before and after treatment with RNase H and oligo(dT).

Given its roles in RNA QC pathways, we next tested whether the nuclear exosome is required for PABPN1-mediated decay. We used siRNAs to knockdown both catalytic cofactors of the nuclear exosome, RRP6 and DIS3 (Figure 4A). Using our transcription pulse-chase assay, we found that PANΔENE RNA was stabilized upon co-depletion of RRP6 and DIS3 (Figure 4B, 4C). Individual knockdowns had only marginal or no effect on PANΔENE stability (Figure S4A), consistent with prior reports of functional redundancy between RRP6 and DIS3 [49]–[51]. In addition, different combinations of RRP6 and DIS3 siRNAs also stabilized PANΔENE RNA, diminishing concerns of off-target effects of siRNAs (Figure S4C).

If PABPN1 stimulates polyadenylation by PAPα or PAPγ as a prerequisite to exosome-mediated decay, then knockdown of RRP6 and DIS3 should lead to the accumulation of hyperadenylated (∼150 to ∼400 nt) RNAs. Indeed, upon exosome depletion, the poly(A) tails were equivalent in length to those from siControl treated cells (Figure 4B and Figure S4B), rather than the shorter forms seen upon PABPN1 or PAPα/PAPγ depletion. Interestingly, upon overexposure, a subset of transcripts migrated as broad heterogeneous smears in the RRP6 and DIS3 depleted cells (Figure 4B). Following treatment with RNase H and oligo(dT), the low mobility smears disappeared (Figure 4D), verifying that the increase in size was due to excess polyadenylation. Because these extremely hyperadenylated (>400 nt) forms were not seen in the control samples, they are unlikely to be natural decay intermediates. Rather, these abnormally long tails may be an experimental artifact of exosome depletion due to the decoupling of polyadenylation and exosome-mediated degradation. Taken together, these data support a role for the exosome in rapid PANΔENE decay. In addition, they are consistent with the model that poly(A) tail extension precedes PANΔENE decay.

### Rapid decay of intronless β-globin requires canonical PAP and exosome activity

**Figure 5:**
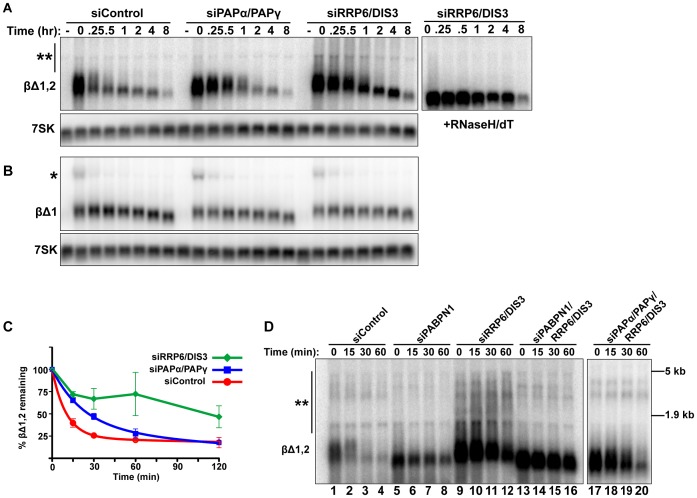
Intronless β-globin decay requires canonical PAP and exosome activity. (**A** and **B**) Representative blot of a transcriptional pulse assay with βΔ1,2 or βΔ1 reporters from cells transfected with the indicated siRNAs. *Right*, Samples from siRRP6/DIS3 lanes were treated with RNase H and oligo (dT). Single asterisk marks the β-globin pre-mRNA and double asterisk denotes the extremely hyperadenylated RNAs. (**C**) Decay curves of the intronless β-globin reporter data from cells transfected with the indicated siRNAs (*n = 3*). The siDIS3/siRRP6 data were not well fit by two-component exponential decay regressions and are represented as linear interpolations (**D**) Results from a transcription pulse assay of βΔ1,2 from cells transfected with the indicated siRNAs. Blot was overexposed to reveal the extremely hyperadenylated RNAs (double asterisks). The relative mobility and sizes of the large and small ribosomal subunits are indicated on the right.

To examine whether the canonical PAPs and exosome were required for mRNA QC, we co-depleted PAPα and PAPγ or RRP6 and DIS3 and monitored intronless β-globin RNA stability. Consistent with the PANΔENE results, intronless β-globin was stabilized upon depletion of these factors (Figure 5A, 5C). In addition, exosome depletion resulted in the accumulation of low mobility transcripts (Figure 5A, double asterisks) due to excessive hyperadenylation, as confirmed by RNaseH/dT analysis (Figure 5A, right panel). In contrast to the intronless reporter, spliced β-globin stability and poly(A) tail length were largely unchanged by either knockdown (Figure 5B). If PABPN1 and the canonical PAPs hyperadenylate RNAs to target them for exosome-mediated decay, then the hyperadenylation observed upon RRP6 and DIS3 co-depletion should be lost in the absence of PABPN1 or the PAPs. Indeed, exosome depletion no longer led to the accumulation of hyperadenylated or extremely long poly(A) tails when PAPs or PABPN1 were co-depleted (Figure 5D, compare lanes 13–16 and 17–20 to lanes 9–12; see Figure S5A for quantification). Similar results were observed for PANΔENE (Figure S5B). Taken together, these data support the conclusion that PABPN1 promotes PAPα or PAPγ-dependent hyperadenylation of nuclear polyadenylated RNAs leading to their degradation by RRP6 or DIS3.

### PABPN1 is responsible for the hyperadenylation of newly made transcripts in vivo

**Figure 6:**
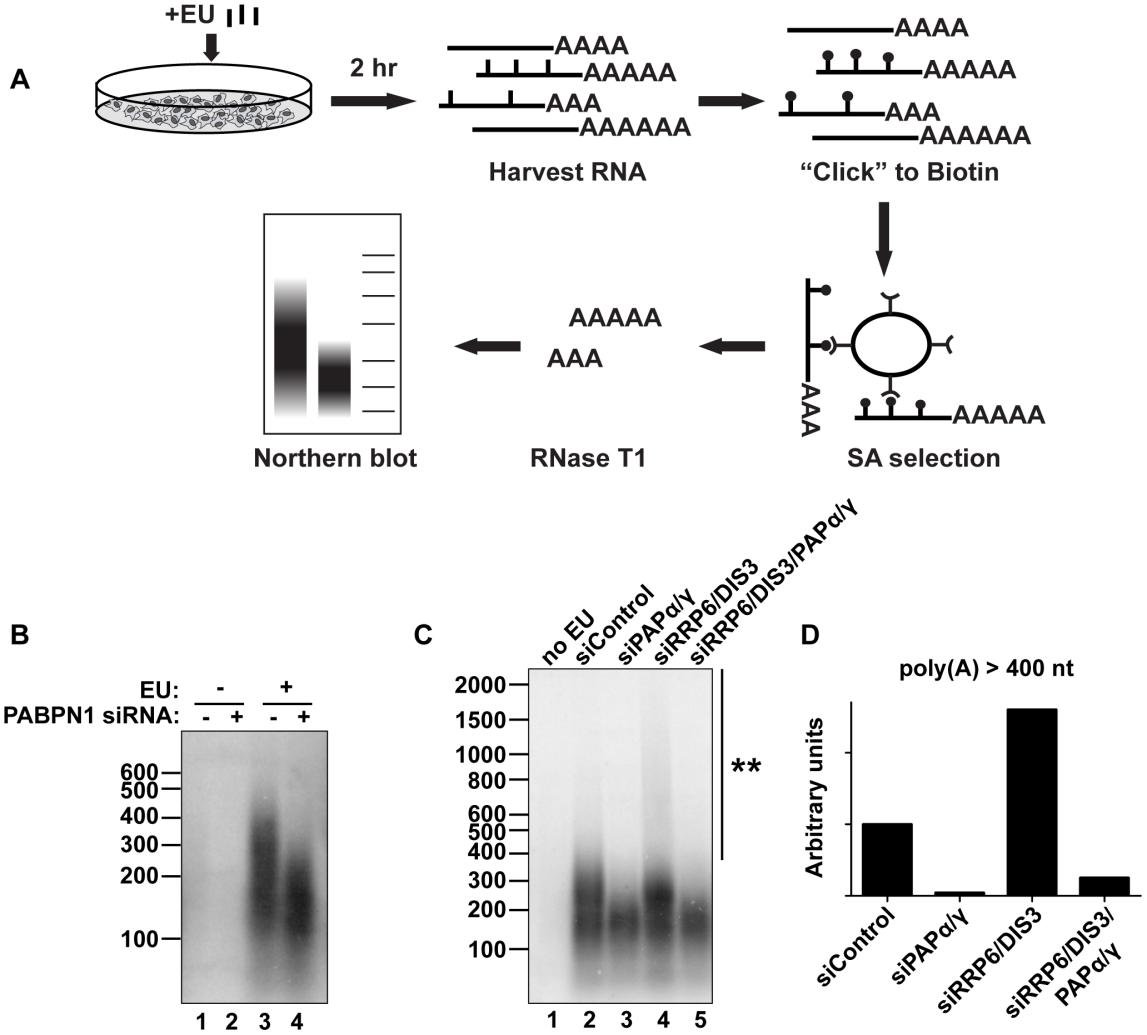
Endogenous transcripts are subject to PABPN1-mediated hyperadenylation. (**A**) Schematic diagram of the in vivo labeling procedure. See text for details. (**B**) Bulk poly(A) tail analysis of labeled RNAs from cells transfected with indicated siRNAs. Prior to harvesting the RNA, cells were incubated with EU for 2 hr as indicated. (**C**) In vivo labeling experiment with cells transfected with the indicated siRNAs. Double asterisk marks extensive hyperadenylation observed upon RRP6 and DIS3 co-depletion. (**D**) Quantification of the poly(A) tails greater than 400 nt in length from (**C**). We “boxed” signals corresponding to poly(A) tails >400 nt, subtracted background signal (no EU) and normalized to the signal from the siRNA control samples.

We next evaluated the impact of PABPN1 on bulk cellular polyadenylated RNAs. To ensure that we monitored RNAs synthesized after functional knockdown of each factor and thereby diminish ambiguities that arise from long-lived transcripts generated prior to knockdown, we developed an in vivo RNA labeling technique to monitor newly made poly(A) tails [52] (Figure 6A). The cells were treated for two hours with the modified nucleoside 5-ethynyluridine (EU), which is efficiently incorporated into transcripts by elongating RNA polymerases [53]. After harvesting cells and RNA extraction, we biotinylated the labeled transcripts with “click” chemistry and purified labeled RNA on streptavidin (SA) beads. The selected RNAs were digested to completion with RNase T1, a G-specific endonuclease that digests total RNA, but leaves the poly(A) tails intact. The resulting poly(A) tails were detected by northern blot using a radiolabeled oligo(dT) probe. Importantly, no poly(A) tails were detected in the absence of EU treatment (Figure 6B, lanes 1 and 2), confirming the specificity of our purification. Cells treated with a control siRNA displayed a broad poly(A) tail distribution (∼100–400 nt) (lane 3) whereas PABPN1 depletion led to the selective loss of the longest poly(A) tails (>∼200 nt) (lane 4). These data mimic what we observed with PANΔENE and intronless β-globin transcripts in that hyperadenylation did not occur when PABPN1 was depleted. However, it remains formally possible that the RNAs with longer poly(A) tails disappeared due to their degradation. Similar results were observed with shorter EU pulses, although the degree of hyperadenylation in the control cells was slightly diminished (Figure S6A). When we collected nuclear and cytoplasmic fractions after the EU pulse, the longer poly(A) tails were primarily observed in the nuclear fraction, as expected if the RNAs are hyperadenylated in a PABPN1-dependent manner (Figure S6B).

We also tested other factors involved in PANΔENE and intronless β-globin rapid decay in this assay. Similar loss of long poly(A) tails was observed when PAPα and PAPγ were co-depleted (Figure 6C, lanes 2 and 3). We additionally found that RRP6 and DIS3 co-depletion increased the accumulation of transcripts with hyperadenylated tails, (compare lanes 2 and 4), particularly those with very long poly(A) tails (>400 nt) (Figure 6D). Importantly, this hyperadenylation was lost if PAPα and PAPγ were co-depleted with the exosome subunits (Figure 6C, lanes 4 and 5, Figure 6D). This response of bulk poly(A) tails to PABPN1, PAP, and exosome depletion closely matches our observations with PANΔENE and intronless β-globin. These data suggest that PABPN1, PAPs, and the exosome are active in the hyperadenylation and decay of a subset of endogenous human transcripts.

**Figure 7:**
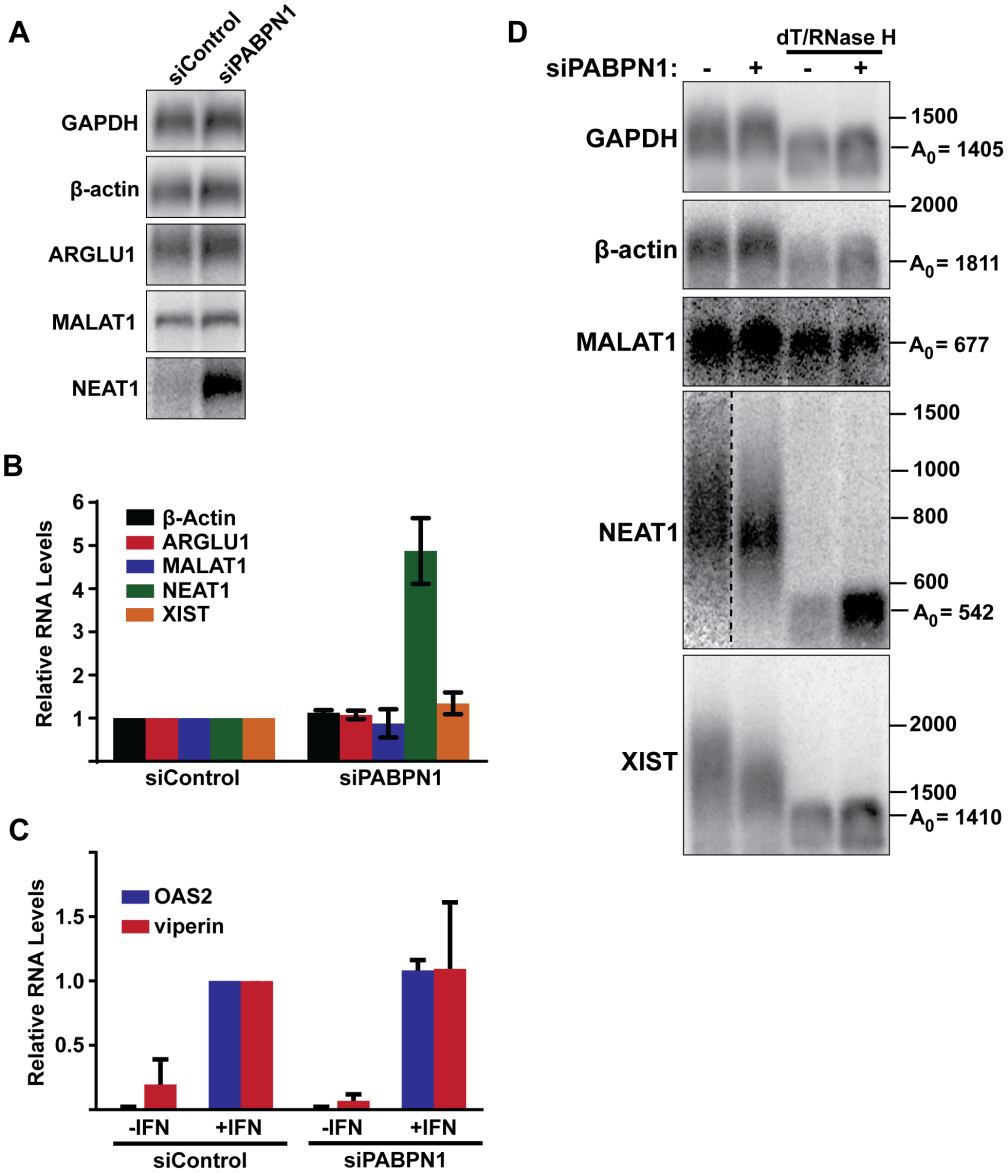
Effects of PABPN1 knockdown on steady-state levels and poly(A) tail lengths of endogenous RNAs. (**A**) Northern blot analysis of several endogenous lncRNAs and mRNAs after siRNA transfection. (**B**) Quantification of the relative abundance of endogeneous transcripts following PABPN1 depletion. All transcripts were quantified from northern blots shown in (A) except XIST, which was quantified from experiments shown in (D), because it is too large to detect by standard northern blot. GAPDH was used for normalization. (*n = 3*) (**C**) Quantitative RT-PCR analysis showing the relative induction of two interferon stimulated genes following PABPN1 depletion and the addition of interferon (IFN). Samples were quantified relative to the siControl/+IFN samples. GAPDH was used as a loading control (*n = 3*). (**D**) Poly(A) tail length analysis of endogenous mRNAs and lncRNAs. Due to their large size, MALAT1, NEAT1, and XIST were cleaved with RNase H and a DNA oligo complementary to a region the near the 3′ end of each RNA. Samples were also treated with RNase H and oligo(dT) as indicated.

To investigate the role of PABPN1 on specific endogenous RNAs, we first examined the effects of PABPN1 depletion on the levels of several mRNAs and nuclear lncRNAs. Upon depletion of PABPN1, the abundance of GAPDH, β-actin, or ARGLU1 mRNAs was unaffected (Figure 7A and 7B). In addition, the nucleocytoplasmic distribution of GAPDH or β-actin mRNAs was unaltered by PABPN1 knockdown (Figure S7A and S7B). These mRNAs were examined at steady state, so it is formally possible that pre-existing, stable mRNAs may mask a general effect of PABPN1 knockdown on mRNA synthesis or decay. To test this, we knocked down PABPN1 and then induced the expression of two interferon stimulated genes, OAS2 and viperin, by adding interferon-α to the cells for 5 hr. Consistent with the steady-state analysis, PABPN1 knockdown had no effect on the accumulation of either IFN-stimulated mRNA (Figure 7C).

Next, we examined the steady-state levels of three abundant and well-studied nuclear lncRNAs, MALAT1, NEAT1, and XIST. Each of these RNAs undergoes a slightly different maturation process. The MALAT1 RNA has no introns, and its 3′ end is produced by RNase P-mediated endonucleolytic cleavage and stabilized by formation of a triple helix structure [54]–[56]. As a result, MALAT1 lacks a poly(A) tail, and thus should not be subject to PABPN1-mediated decay. NEAT1 is also intronless, but it possesses a conventional poly(A) tail. In this sense, NEAT1 is most analogous to our PANΔENE reporter RNA. Finally, XIST is polyadenylated by the canonical cleavage and polyadenylation machinery, but has multiple introns. PABPN1 depletion had distinct effects on the levels of each of these nuclear noncoding RNAs (Figure 7A and 7B). We saw no significant changes in MALAT1 or XIST lncRNA levels (Figure 7A and 7B). In marked contrast, NEAT1 levels increased ∼5-fold when PABPN1 was depleted. These results are in agreement with a recent study showing that the steady state levels of most mammalian mRNAs are unaffected upon PABPN1 knockdown but that a subset of nuclear noncoding RNAs, including NEAT1, increased in abundance [57].

We also examined the relative poly(A) tail lengths of several transcripts in the presence or absence of PABPN1. Knockdown of PABPN1 had no effects on the mobility of GAPDH mRNA, β-actin mRNA, or the MALAT1 lncRNA (Figure 7D). However, upon PABPN1 depletion, the mean poly(A) tail lengths of NEAT1 were shorter and less heterogeneous than in the control cells, mirroring our observations with PANΔENE and intronless β-globin. Interestingly, in the case of the XIST lncRNA, the mean poly(A) tail distribution was shorter in the absence of PABPN1 (Figure 7D), but the steady-state levels increased only marginally (∼30%) (Figure 7B). Thus, it appears that PABPN1-dependent nuclear hyperadenylation can be separated from the subsequent decay pathways in specific cases (see Discussion). These results are consistent with the reporter assays and support the conclusion that PABPN1 can promote the hyperadenylation and decay of endogenous nuclear transcripts.

## Discussion

Despite significant progress in uncovering the mechanisms of nuclear RNA decay in yeast, these processes remain relatively unexplored in higher eukaryotes. In this study, we used a viral nuclear RNA and intronless β-globin reporters to identify components of a rapid human nuclear RNA decay pathway. Through a targeted knockdown approach, we identified PABPN1, the canonical PAPs, PAPα and PAPγ, and the nuclear exosome components RRP6 and DIS3 as central players in this rapid decay pathway. We propose that this pathway promotes the decay of nuclear mRNAs undergoing RNA QC and polyadenylated nuclear noncoding RNAs.

### Poly(A) tail extension and nuclear RNA decay

The relationship between RNA polyadenylation, hyperadenylation and RNA decay is complex. Hyperadenylated RNAs are often observed when RNA decay factors or other RNA processing factors are compromised, but it is difficult to empirically determine whether the hyperadenylation promotes RNA decay or if hyperadenylated transcripts appear as a consequence of manipulating nuclear RNA metabolism. Several of our observations suggest that poly(A) tail extension is mechanistically linked to decay. Knockdown of either PABPN1 or co-depletion of the PAPs leads to more stable RNAs with shorter poly(A) tails. Importantly, the tails observed upon PABPN1 knockdown are not completely lost, but are still ∼50–150 nt for PANΔENE RNA (Figure 1F) and ∼100–200 nt in the case of newly made bulk poly(A) RNA (Figure 6). Overexpression of the PABPN1 LALA mutant, which binds RNA but cannot stimulate polyadenylation, strongly stabilized PANΔENE RNA and led to shorter poly(A) tails (Figure 3F, 3G). Thus, PABPN1-dependent extension of the poly(A) tails to lengths longer than ∼200 nt may precede decay. Consistent with this, exosome depletion led to the stabilization of hyperadenylated transcripts, some with very long tails, and generation of hyperadenylated tails depends on PABPN1 and the PAPs (Figures 4 and 5). In addition, when examined shortly after transcription shut-off (t = 15 min), we sometimes observed that PANΔENE RNA became slightly longer immediately prior to its destruction (for example, compare lanes 2 and 3 in Figure 1B) suggesting we were able to detect the poly(A) tail extension prior to transcript decay. Finally, inhibition of poly(A) tail extension by cordycepin inhibited transcript decay, even when cordycepin was added coincident with transcription shut-off (Figure 3H and 3I). Importantly, a terminal 3′deoxynucleotide has no effect on exosome activity in vitro (C. Lima, personal communication), supporting our interpretation that the stability effects seen here are due to the inhibition of polyadenylation, rather than exonucleolytic decay directly. Admittedly, we cannot completely rule out indirect effects of cordycepin on RNA metabolism. However, when taken together, our data are most consistent with the model that poly(A) tail extension is directly involved with transcript decay and RNA QC.

**Figure 8:**
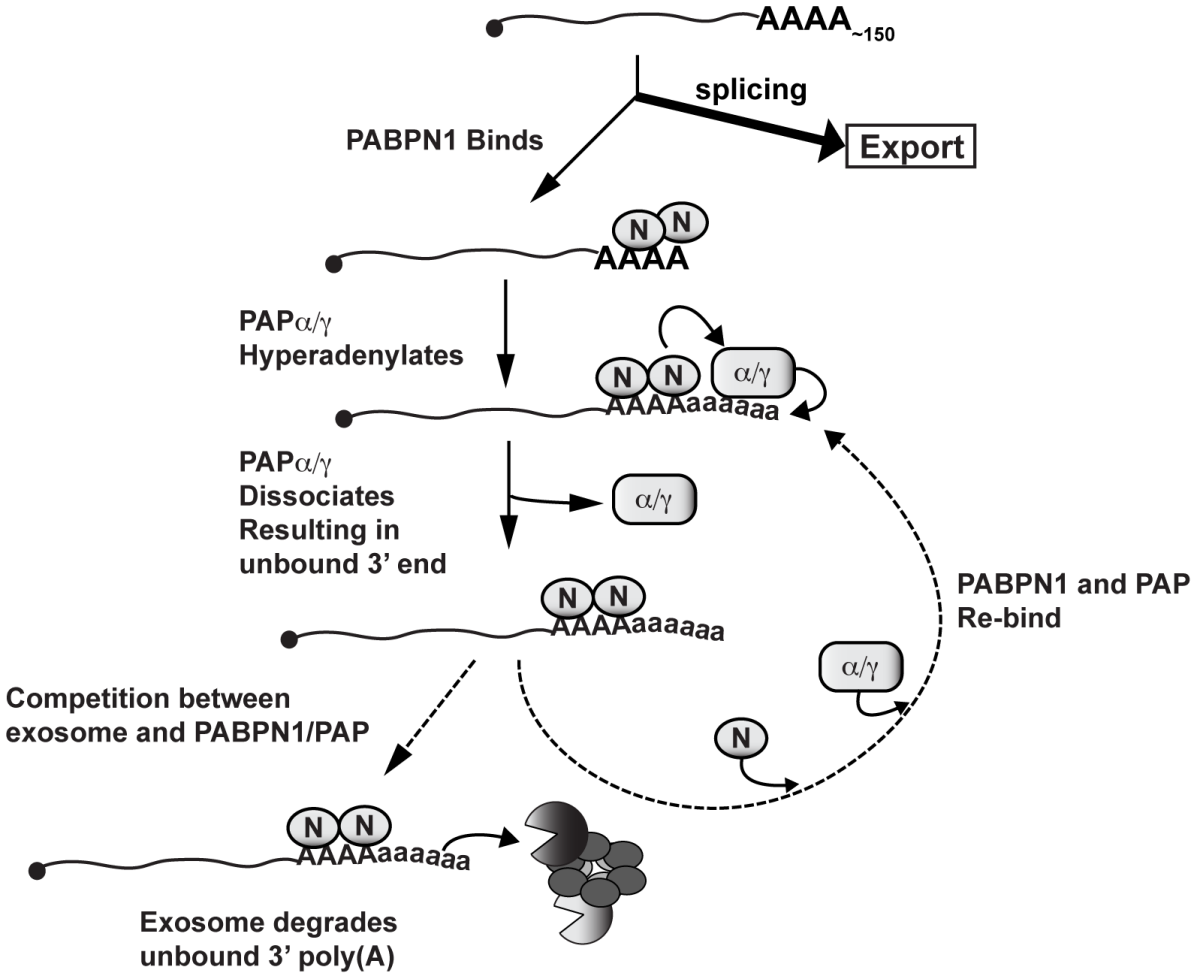
Model of PABPN1-mediated decay. Diagram depicting a speculative mechanism of PABPN1-mediated decay. Symbols: PABPN1 (oval inscribed with “N”), PAPα/PAPγ (rectangle inscribed with “α/γ”), RRP6 and DIS3 (pacman symbols), core exosome (ring of ovals), lower case “a” in poly(A) tails denotes hyperadenylation. Note that PABPN1 is not depicted on the initial poly(A) tail for simplicity and is not meant to imply any mechanisms for PABPN1 in the initial polyadenylation event (see Discussion).

How might PABPN1 and PABPN1-dependent hyperadenylation be linked to RNA decay? PABPN1 and PAPs may provide the exosome a suitable binding site by stimulating the polyadenylation of a “naked” poly(A) tail (Figure 8). Recent biochemical and structural studies showed that ∼30 nt of naked RNA is required to bind the exosome central channel in order to productively engage DIS3 for processive exosome degradation [58]–[60]. Poly(A) tail extension may be required to transiently produce unbound poly(A) 3′ ends sufficiently long enough to extend through the exosome channel. In this model, poly(A) tail extension leads to a kinetic competition between additional PABP binding (PABPN1 itself or other PABPs) and exosome binding. When the exosome wins the competition, its processive activity will lead to rapid RNA destruction. If PABPN1 and PAP re-bind, the process repeats itself. In contrast, if other PABPs bind, the RNA may exit this cycle and proceed to normal function (not depicted in Figure 8). Previous in vitro studies showed that PABPN1 contributes to distributive polyadenylation by PAP (see below), our model proposes that this function serves to regulate RNA decay in the cell nucleus.

PABPN1 may further contribute to decay by direct recruitment of the exosome. The exosome has relatively weak affinity for poly(A) RNA [58], and may require “bridging” factors in order to efficiently degrade RNA. In fact, *S. pombe* Pab2 appears to function in this fashion (see below). Notably, PABPN1 has been reported to physically interact with RRP6 and RRP40 [57], further supporting this mechanism. Additional studies are needed to test these models and determine the detailed molecular mechanisms connecting PABPN1 stimulation of hyperadenylation and exosome recruitment with rapid RNA decay.

In an alternative model, PABPN1 and PAP activities do not directly stimulate decay but are essential to “license” the RNA for degradation. In this case, PABPN1 and PAP-mediated polyadenylation is a necessary upstream step in RNA biogenesis. Prior to the completion of this step, RNAs are resistant to decay by the exosome, perhaps due to subcellular localization or RNP structure. When PAPs or PABPN1 are knocked down, the RNA does not progress from this state and therefore the transcript has a longer half-life. Several aspects of this model are similar to yeast systems in which accumulation of the RNAs at the site of transcription is coupled with RNA QC [9], [10]. Furthermore, recent studies have shown that the NPM1 protein is deposited on mRNAs as a result of proper termination of polyadenylation [61], [62]. It is tempting to speculate that NPM1 deposition is the molecular licensing signal. However, the licensing model predicts that most transcripts would be subject to the observed PABPN1-mediated hyperadenylation as part of their normal biogenesis. In contrast, under conditions in which we see robust effects on our reporters and NEAT1, we see little effect of PABPN1 knockdown on the cytoplasmic accumulation of β-actin, GAPDH, or bulk mRNAs (Figure S6B and Figure S7).

### An evolutionarily conserved role for nuclear poly(A) binding proteins in RNA decay

The present work complements recent studies in both fission and budding yeast pointing to novel roles for nuclear PABPs in RNA decay. In budding yeast, the nuclear poly(A)-binding protein, Nab2, binds to hyperadenylated tails generated by TRAMP and recruits Rrp6 to degrade the transcript [14]. Additionally, Nab2 autoregulates the *NAB2* mRNA by promoting Rrp6-mediated decay [63], [64]. However, Nab2 is not a homolog of PABPN1 and no *S. cerevisiae* PABPN1 homolog has been described [65]. Moreover, Nab2 does not stimulate yeast PAP activity in vitro and loss of Nab2 leads to hyperadenylation of bulk transcripts rather than mirroring the loss of long poly(A) tails observed with PABPN1 depletion (Figure 6) [36], [66], [67]. Nab2 homologs have been identified in metazoans [68], [69], but their functions remain poorly understood. Therefore, it remains unclear whether Nab2 is an appropriate model for exploring PABPN1 function.

Perhaps a better model for human PABPN1 is the fission yeast Pab2 protein, which shares 47% identity and 66% similarity with PABPN1 [35]. Deletion of Pab2 stabilizes pre-snoRNAs, a subset of pre-mRNAs, and meiosis-specific mRNAs [31]–[34], [70]. Similar to our results, Pab2-mediated decay requires polyadenylated targets, the canonical PAP (Pla1), and Rrp6. However, there are important mechanistic distinctions between PABPN1 and Pab2 in decay. For example, while human PABPN1 stimulates PAP processivity, Pab2 does not stimulate Pla1 in vitro [28]. Most strikingly, deletion of Pab2 or PABPN1 has opposing effects on poly(A) tail lengths. Deletion of Pab2 leads to increases in bulk poly(A) lengths and to the hyperadenylation of at least some of its targets [32]–[35], whereas PABPN1 depletion decreases bulk poly(A) tail lengths (Figure 6) [38]. Interestingly, the *Drosophila* PABPN1 homolog mimics the human PABPN1 in that it stimulates its cognate PAP and its depletion leads to poly(A) tail shortening [37], [71], [72], suggesting that these functions may be restricted to metazoans. As described above, we favor a model in which PABPN1-mediated stimulation of polyadenylation constitutes an important part of a nuclear RNA decay pathway and PABPN1 may additionally recruit the exosome. In contrast, the work in *S. pombe* suggests the primary function of Pab2 in decay is to recruit the exosome to polyadenylated transcripts. Upon Pab2 deletion, the exosome is less efficiently recruited, resulting in increased Pla1 activity and hyperadenylation. Thus, while nuclear poly(A)-binding proteins from fission yeast, budding yeast, and humans all promote nuclear RNA decay, the mechanistic details appear to differ considerably.

### Targets of PABPN1-mediated RNA decay

A recent RNA-seq study demonstrated that PABPN1 depletion had little effect on the levels of most transcripts, but a subset of nuclear lncRNAs were stabilized [57]. These results are entirely consistent with our data with PAN RNA, a viral lncRNA. In addition, we propose this pathway targets export-deficient mRNAs, similar to our intronless β-globin mRNA. These transcripts would remain undetected in RNA-seq studies because they will represent only a small subpopulation of any specific mRNA. Interestingly, a large fraction of newly made RNAs acquire long poly(A) tails in a PABPN1-dependent fashion (Figure 6B). If these transcripts all represent (pre-)mRNAs targeted for elimination by RNA QC pathways, then it is noteworthy that the cell generates so many aberrant RNAs. Moreover, it seems unlikely that these transcripts are primarily lncRNAs as these are generally low abundance transcripts. Alternatively, some of the newly made RNAs with long poly(A) tails may be hyperadenylated in a manner that is uncoupled from the subsequent decay processes. In fact, this appears to be the case for XIST, as we observed shorter poly(A) tails upon PABPN1 depletion, but the steady-state levels increased only marginally (Figure 7B, 7D). Interestingly, PANΔENE, intronless β-globin and NEAT1 are all single-exon transcripts, whereas XIST has multiple exons, consistent with previous reports indicating a mechanistic link between splicing factors and RNA stability [24], [41], [73]–[75]. Alternatively, XIST and other stable polyadenylated nuclear RNAs may have evolved other cis-acting mechanisms to promote RNA stability. Indeed, wild-type PAN RNA uses the ENE for protection from nuclear decay factors. Future experimentation will focus on determining the identity and regulation of these abundant PABPN1-mediated hyperadenylated transcripts.

### The role of PABPN1 in the initial polyadenylation event

PABPN1 and PAPα are generally thought to be required for the initial polyadenylation of nascent transcripts. However, under conditions in which we observed effects on hyperadenylation and RNA stability of our reporters and the endogenous NEAT1, we see little or no effect of PABPN1 knockdown on steady-state mRNA levels, mRNA nucleocytoplasmic distribution or in the accumulation of IFN-stimulated mRNAs. Moreover, changes in poly(A) tail lengths of spliced β-globin reporters or bulk newly-made cytoplasmic poly(A) tails were not as large as those observed for nuclear RNAs. There are several possible interpretations of our seemingly contradictory data. First, PABPN1 and PAP are required for both initial polyadenylation and subsequent hyperadenylation, but our knockdown was sufficient only to affect hyperadenylation. Because the PAPs are catalytic factors, low amounts may be sufficient for activity. Additionally, low levels of PABPN1 can stimulate PAPs in vitro [76], so it is possible that the PABPN1 remaining after knockdown could provide function. Second, the distributive PAPα activity observed in the absence of PABPN1 may be sufficient to produce the poly(A) tails observed over a two-hour transcription pulse. Importantly, our assays do not measure the rate of the initial polyadenylation reaction, so it is possible that there is a reduced rate of initial PAP activity in the absence of PABPN1, but it is beyond our experimental parameters to measure it. However, even though the poly(A) tails reach lengths of ∼50–150 nt during the 2-hr transcription pulse, they are not extended during the subsequent 2-hr chase (Figure 1, lanes 9–14), suggesting that the shorter poly(A) tails are not simply the result of slower polyadenylation or that further extension is counterbalanced by deadenylases. Third, other proteins may substitute for PABPN1 in its absence. Indeed, a recent study found that when PABPN1 is depleted, the cytoplasmic poly(A) binding protein PABPC4 is relocalized to the nucleus where it associates with the polyadenylation machinery [77]. Fourth, PABPN1-mediated stimulation of PAP processivity may not be absolutely required for the formation of initial poly(A) tails in vivo. The evidence that PABPN1 participates in the initial cleavage and polyadenylation event is derived from in vitro experiments. These experiments show that PABPN1, synergistically with the cleavage and polyadenylation specificity factor (CPSF), promotes processive PAPα activity in the initial polyadenylation reaction [28]. Once the tail reaches ∼250 nt, PABPN1 alone is largely required for further polyadenylation [46], [76]. Therefore, perhaps CPSF alone or with other unknown factors is sufficient to promote PAPα processivity during the primary mRNA 3′-end formation in vivo. Importantly, even if PABPN1 is not required for the initial polyadenylation reaction, our results are remarkably consistent with the in vitro evidence supporting a role for PABPN1 in promoting polyadenylation beyond ∼250 nt. Distinguishing among these possibilities will provide important insight into the complex roles of nuclear polyadenylation and poly(A)-binding proteins in mRNA biogenesis and decay.

## Materials and Methods

### Plasmids and cell culture

The TetRP-driven PANΔENE and β-globin reporter constructs were described previously (TRP-PANΔ79) [23], [24]. The myc-PABPN1 overexpression construct was a gift from Dr. Lynne Maquat (University of Rochester) [78]. Point mutations were generated by PCR using myc-PABPN1 as a template. PCR fragments were cloned into myc-PABPN1 to generate myc-tagged LALA. Positive clones were confirmed by sequencing. 293A-TOA cells [39] were grown in Dulbecco's modified Eagle's medium (Sigma) containing 10% Tetracycline-free fetal bovine serum (Clontech), 1× penicillin/streptomycin (Sigma), 2 mM L-glutamate, and 100 µg/mL G418.

### Transfections

Cells were transfected with 10 nM siRNA (Silencer Select, Ambion; Table S2) using RNAiMAX transfection reagent (Invitrogen) according to the manufacturer's instructions. One day following transfection, ∼100% confluent cells were diluted to new plates to allow the cells to divide for an additional 24–48 hours, after which they were transfected with the appropriate reporter constructs. For DNA transfections, cells were transfected with TransIT-293 (Mirus) with the following modifications to the manufacturer's protocol. For transfection of a single well from a 12-well plate, 0.5 µL TransIT-293, 10 µL Opti-MEM reduced serum media (Gibco), and 0.2 µg of DNA were used. A typical transfection consisted of 0.1 µg pcDNA3 and 0.1 µg reporter construct. Twenty-four hours following transfection cells were harvested using TRI Reagent (Molecular Research Center).

### RNase H analysis

Total RNA (1–3 µg) was incubated at 37°C for 1 hour in a 20 µL mixture containing 0.3 U RNase H (Promega), 20 U RNasin (Promega), 300 ng poly(A) RNA (Sigma), 10 mM dithiothreitol, 100 mM KCl, 10 mM MgCl_2_, and 20 mM Tris-HCl (pH 7.0), and plus or minus 0.5 µM dT_40_. We found that the addition of poly(A) was necessary to counter a trace deadenylation activity present in the reaction. In Figure 2, internal cleavage of PANΔENE RNA was directed by the addition of 0.5 µM NC30 (5′ CAATGTTCTTACACGACTTTGAAACTTCTGACAAATGCC 3′). Following digestion, 180 µL of G50 buffer (0.25% SDS, 0.3 M NaOAc, 20 mM Tris pH 6.8, and 2 mM EDTA) was added to stop the reaction. After PCA extraction, the RNA was precipitated in 70% ethanol and run on a 1.6% agarose gel alongside a 0.1 to 2 kb RNA ladder (Invitrogen). Blots were probed with an end-labeled oligo probe specific to the 3′ end of PANΔENE RNA. Poly(A) tail lengths were estimated by comparison with the size marker. For analysis of endogenous transcripts 90 µg total RNA was treated with RNase H and DNA oligos complementary to the 3′ ends of MALAT1 (5′ AAGCACCGCTTGAGATTTGGG 3′), NEAT1 (5′ TTCCAAACTGATTTTAGGTGA 3′), and XIST (5′ TAGACAAACCTTGTAAATGC 3′). The final concentration of each DNA oligo was 0.5 µM in a 60 µL reaction. Following RNase H treatment, RNA was poly(A) selected according to standard protocols and half of each sample was treated with RNase H and oligo(dT) and the 3′ cleaved fragments were detected by northern blot.

### Transcription pulse-chase assays

Transcription pulse-chase assays were performed as previously described [24], [39] with the following exceptions. Twenty-four hours post-siRNA transfection, cells were plated on 12-well plates. Twenty-four to 48 hours later, depending on the targeted mRNA, cells were transfected with the appropriate DNA constructs. Cells were grown in the presence of 5 ng/mL of dox (Sigma-Aldrich) to repress transcription. Twenty-four hours after DNA transfection, cells were washed twice with 1× Dulbecco's Phosphate Buffered Saline (PBS) with calcium/magnesium (Sigma-Aldrich), and cells were incubated in dox-free media for two hours. Dox was added to a final concentration of 50 ng/mL to repress transcription. For the experiments in Figure 1 and S1, 20 µg/mL cordycepin (Sigma-Aldrich) was added upon initiation of the two-hour pulse and remained in the media after dox addition. Total RNA was harvested at the appropriate times using TRI Reagent, and analyzed by northern blot. Membranes were probed with radiolabeled antisense transcripts for PAN RNA or β-globin, and subsequently for 7SK RNA.

### Regression analysis

We fit transcription pulse-chase data to two-component exponential decay curves with Prism 5 software (GraphPad) using default parameters with two additional constraints. First, the decay constant for the rapid decay (K_fast_) pathway was set to ≥0.046 corresponding to a t_1/2_≤15 [24]. Second, we set the plateau value to zero. While all the replicates were used in regression analysis (n≥3), the reported R^2^ values (Table S1) are derived from the fit of the mean values only. To focus on the rapid decay process, we took samples for up to 2 hrs. Therefore, we report t_1/2_ values for the slow decay pathway as >120 min if they exceed the sampling time. All decay curves were fit using this method, except for the siRRP6/siDIS3 data in Figure 5C and LALA overexpression data in Figure 7D which did not conform to this model and Figures S3D and S3F which had no data points for times <1 hr thereby ruling out the definition of rapid decay pathway.

### Immunoblotting

Protein was harvested using TRI Reagent according to the manufacturer's instructions. Protein was separated on SDS-PAGE and western blotted using standard procedures. The following antibodies were used: anti-PABPN1 (Abcam; ab75855), anti-PAPα (Abcam; ab126934), anti-PAPγ (Novus Biologicals; NBP1-30061), anti-RRP6 (EXOSC10) (Abcam; ab50558), anti-DIS3 (Bethyl Laboratories; A303-765A), anti-β-actin (Abcam; ab6276). Quantitative westerns were performed using infrared detection with an Odyssey Fc and quantification was performed using ImageStudio software (LI-COR Biosciences).

### Metabolic labeling and selection of newly made RNAs

Click-iT Nascent RNA Capture Kit (Invitrogen) was used with modifications described in [52]. Briefly, two to four days following knockdown, cells were exposed to 200 µM EU for two hours. RNA was harvested using TRI Reagent. EU-containing transcripts were selected from 500 ng of total RNA using the biotinylation and SA selection described in Grammel et al. (2012). For the experiment shown in Figure S6A, three different pulse times (120′, 60′, and 30′) were used. Because longer pulse times would be expected to label more RNA, we increased the amount of RNA used from the shorter pulses (0.5 µg for 120 min, 1 µg for 60 min, and 2 µg for 30 min). For the experiments in Figure S6, a biotinylated DNA oligonucleotide (5′ GATATTGAATCGAAAATCATATCTTTGATAATAGACTACTCAAGACTTTGTCCCGATTCTCCTTTAAACTTGAAG-Btn 3′) was added to the reaction in order to control for differences in recovery. The loading control was detected with a complementary DNA oligo (5′ GTCTTGAGTAGTCTATTATCAAAGATATGATTTTCGATTC 3′).

### Bulk poly(A) RNA analysis

Total or SA-captured biotinylated RNA was incubated at 37°C for 15 minutes in a 30 µL reaction containing 20 mM TrisHCl (pH 6.8), 8 U RNasin and 150 U RNase T1 (Ambion). The reaction was stopped by adding 170 µL G50 buffer containing 0.1 mg/mL Proteinase K (Fisher) and incubated at 37°C for 30 minutes. After PCA extraction and ethanol precipitation, products were separated on a 1.8% agarose-formaldehyde gel, and analyzed by northern blot using a dT_40_ probe end-labeled with T4 polynucleotide kinase.

### Northern blotting

Standard northern blotting techniques [79] were used to probe for PAN, β-globin, and 7SK. For the experiments performed in Figure 7, we made several modifications. In order to detect multiple RNAs per gel, we made cleavable riboprobes that could be stripped from the membrane. To do this, we generated probes incorporating a partial phosphorothioate backbone, which can be subsequently cleaved by treatment with iodine. Upon cleavage, the full-length probe is reduced to a number of smaller fragments, which can be easily removed with washing. A typical probe reaction consisted of 40 mM Tris (pH = 7.5) 6 mM MgCl_2_, 4 mM spermidine, 10 mM DTT, 200 ng T7-driven template, 0.5 mM ATP, CTP, and GTP, 2 U/µL RNasin, 50 µM UTTPαS (TriLink Biotechnologies), 25 µCi of α-^32^P-UTP (800 Ci/mmol), and T7 RNA polymerase. After visualizing the target RNA, the membrane was incubated at 65°C for 30 minutes in a solution of 200 mM sodium phosphate buffer (pH = 7.2), 50% formamide, 7% SDS, and 6.25 mM I_2_. Immediately following the cleavage reaction, the probe was stripped by washing the membrane twice in boiling 0.5% SDS.

### Interferon and qPCR

Two days following siRNA transfection, cells were treated for five hours with 100 U/mL human interferon-α A (PBL Interferon Source). RNA was harvested, and cDNA was made with random hexamers as primers using standard molecular biology techniques. Quantitative real-time PCR was performed using iTaq Universal SYBR Green Supermix (BIO-RAD). Primers: GAPDH 5′ AGCCTCAAGATCATCAGCAATG, GAPDH 3′ ATGGACTGTGGTCATGAGTCCTT. Viperin (QT01005256) and OAS2 (QT00005271) primers were obtained from Qiagen.

### Nucleocytoplasmic fractionation

Two days following knockdown, cells from 10 cm plates were trypsinized and resuspended in ice-cold media. Cells were pelleted with gentle centrifugation at 500 g for 3 minutes at 4°C, and then washed with ice-cold PBS. Following the wash step, cells were resuspended in 600 µL of ice cold Buffer I (0.32 M Sucrose, 3 mM CaCl_2_, 2 mM MgCl_2_, 0.1 mM EDTA, 10 mM Tris (pH = 8.0), 1 mM DTT, 0.4 U/µL RNasin, and 0.5% Igepal) and incubated for 5 minutes on ice. The cell lysates were spun at 500 g for 5 minutes at 4°C. The supernatant (“cytoplasmic” fraction) was added to 5 mL TRI Reagent and the pellet (“nuclear” fraction) was resuspended in 600 µL Buffer I before addition of 5 mL TRI Reagent. In order to ensure consistent loading between samples, we poly(A) selected 40 µg of RNA from the cytoplasmic fraction and 10 µg of RNA from the nuclear fraction. To control for the quality of the fractionation, an aliquot of non-poly(A) selected RNA was probed for pre-ribosomal RNA using a DNA oligo probe (5′ AACGATCAGAGTAGTGGTATTTCACC 3′).

## Supporting Information

Figure S1 Related to Figure 1. (**A** and **B**) Quantitative western blot analysis of total lysate from cells transfected with either a control siRNA or an siRNA pool against PABPN1. The blot was probed with antibodies against PABPN1 and β-actin (loading control) and the signal was quantified relative to the β-actin loading control. (**C**) Western blot analysis of protein from the time zero samples in Figure 1E.
(TIF)

Figure S2 Related to Figure 2. Representative transcription pulse-chase of a spliced (βΔ1) reporter over an eight-hour time course.
(TIF)

Figure S3 Related to Figure 3. (**A**) Decay curves of PANΔENE from cells transfected with the indicated siRNAs *(n = 3)*. (**B**) Northern blot analysis of PANΔENE RNA from cells transfected with independent pools of siRNAs targeting PAPα and PAPγ (**C**) Quantitative western blot analysis of total lysate from cells transfected with either a vector control, or LALA PABPN1. The blot was probed with antibodies against either PABPN1 (green) or β-actin (red) as a loading control. The relative abundance of PABPN1 normalized to β-actin is indicated below each lane. Importantly, because these values include protein from untransfected cells, they are likely an underestimate of the degree of PABPN1 overexpression. (**D**) Relative poly(A) tail lengths of PANΔENE RNA following LALA overexpression. RNA from cells treated with siPABPN1 was included for comparison. (**E**) Relative poly(A) tail lengths of PANΔENE RNA following cordycepin treatment. PANΔENE RNA was cleaved with RNase H targeted by NC30 and oligo(dT) as indicated. The cleaved RNA was analyzed by northern blot using a 3′ end-specific probe. We estimated that the poly(A) tails in the presence of cordycepin were between ∼20–50 nt. (**F**) Representative transcription pulse assay of βΔ1,2 from cells induced in the presence or absence of cordycepin. (**G**) Linear interpolation of the results from (C)(*n = 3*). (**H** and **I**) Same as in (**F**) and (**G**) except spliced β-globin (βΔ1) was assayed. The same blot and exposure are shown for both panels in (**H**).
(TIF)

Figure S4 Related to Figure 4. (**A**) Decay curves of PANΔENE from cells transfected with the indicated siRNAs (*n = 3*). (**B**) A comparison of the relative lengths of PANΔENE from cells transfected with the indicated siRNAs. RNA is from the time zero samples (lanes 2 and 10 in Figure 4B). (**C**) Results from a transcript pulse assay of PANΔENE from cells transfected with two independent pools of siRNAs targeting RRP6 and DIS3.
(TIF)

Figure S5 Related to Figure 5. (**A**) Quantitation of the percent of intronless β-globin which was “extremely hyperadenylated” in each lane of Figure 5D. The “% extremely hyperadenylated” was calculated by boxing the signal from each lane above the primary band, subtracting the background signal, and dividing by the total amount of signal in each lane. (**B**) Results from a transcription pulse assay of PANΔENE from cells transfected with the indicated siRNAs. Blot was overexposed to reveal the extremely hyperadenylated RNAs (double asterisks).
(TIF)

Figure S6 Related to Figure 6. (**A**) Bulk poly(A) tail analysis from cells transfected with either siControl or siPABPN1. Prior to harvesting the RNA, cells were incubated with EU for the indicated pulse times. To compensate for the shorter pulse times, increasing amounts of total RNA was used for the click reaction: 0.5 µg for the 120′ pulse, 1 µg for the 60′ pulse, and 2 µg for the 30′ pulse and the no EU sample. An exogenously added biotinylated DNA oligo (“Loading control”) was used to control for recovery. Note that this DNA only controls for RNA recovery and loading during the experimental procedure and does not necessarily reflect the total amounts of input RNA. (**B**) Nuclear/cytoplasmic distribution of poly(A) tails made in the presence or absence of PABPN1. Cells were separated into nuclear and cytoplasmic fractions following a two-hour EU pulse.
(TIF)

Figure S7 Related to Figure 7. (**A**) Northern blot analysis of nuclear and cytoplasmic fractions following PABPN1 depletion. The blot was probed for β-actin and GAPDH, as well as pre-ribosomal RNA in order to control for the quality of the fractionation. The amount of RNA loaded was kept constant at a 4∶1 cytoplasmic∶nuclear ratio. (**B**) Quantification of the results in panel (**A**) (*n = 3*).
(TIF)

Table S1 Kinetic parameters estimated from regression analysis of decay data.
(DOCX)

Table S2 siRNAs used in this study.
(DOCX)
