# Supplementary figures and images for: The Human Nuclear Poly(A)-Binding Protein Promotes RNA Hyperadenylation and Decay
Source: PLoS Genet. 2013 Oct 17;9(10):e1003893. doi: 10.1371/journal.pgen.1003893 (PMC3798265; doi:10.1371/journal.pgen.1003893)

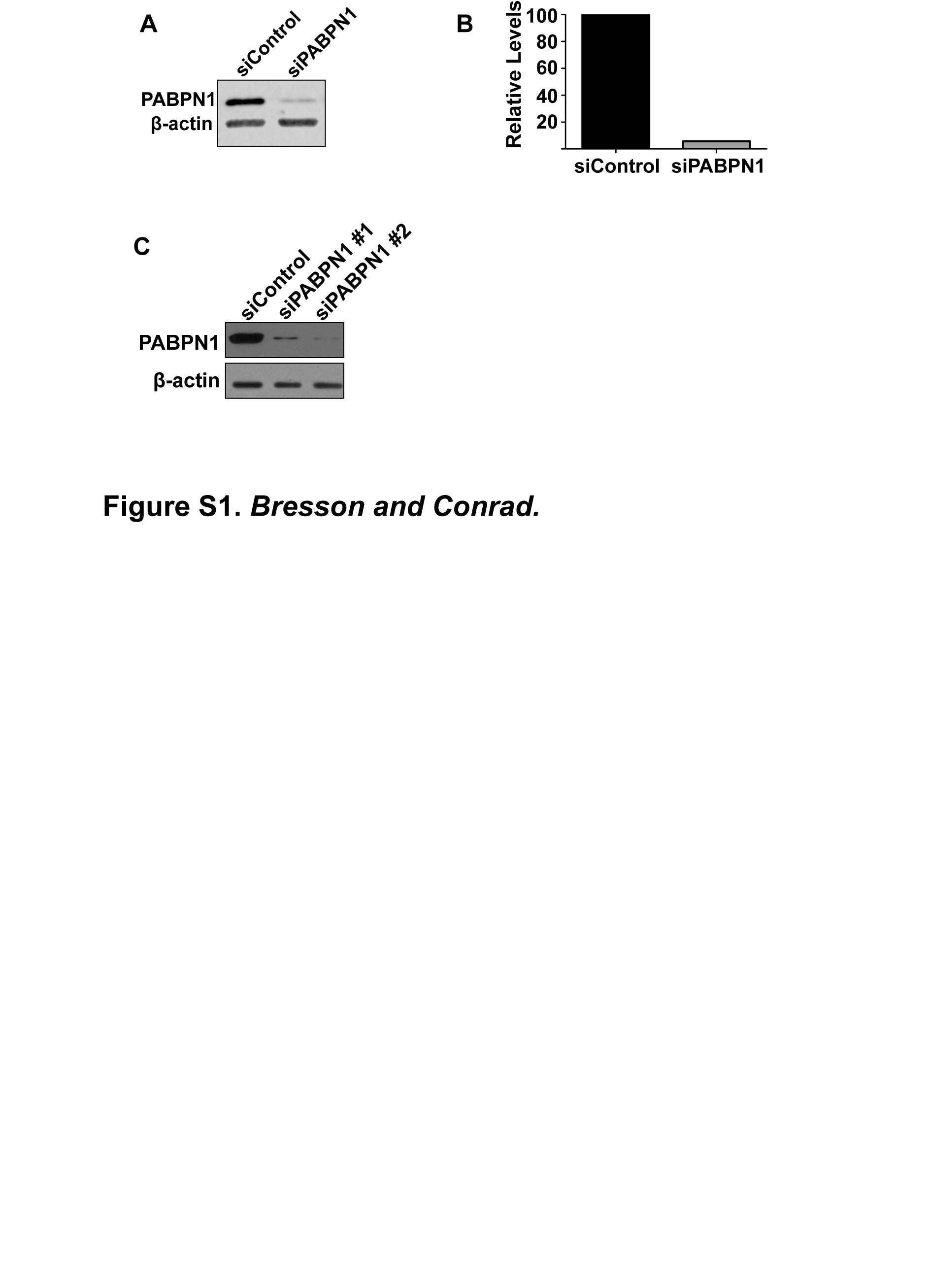

Supplement: Figure S1 — Related to Figure 1. (A and B) Quantitative western blot analysis of total lysate from cells transfected with either a control siRNA or an siRNA pool against PABPN1. The blot was probed with antibodies against PABPN1 and β-actin (loading control) and the signal was quantified relative to the β-actin loading control. (C) Western blot analysis of protein from the time zero samples in Figure 1E. (TIF) [file pgen.1003893.s001.tif]

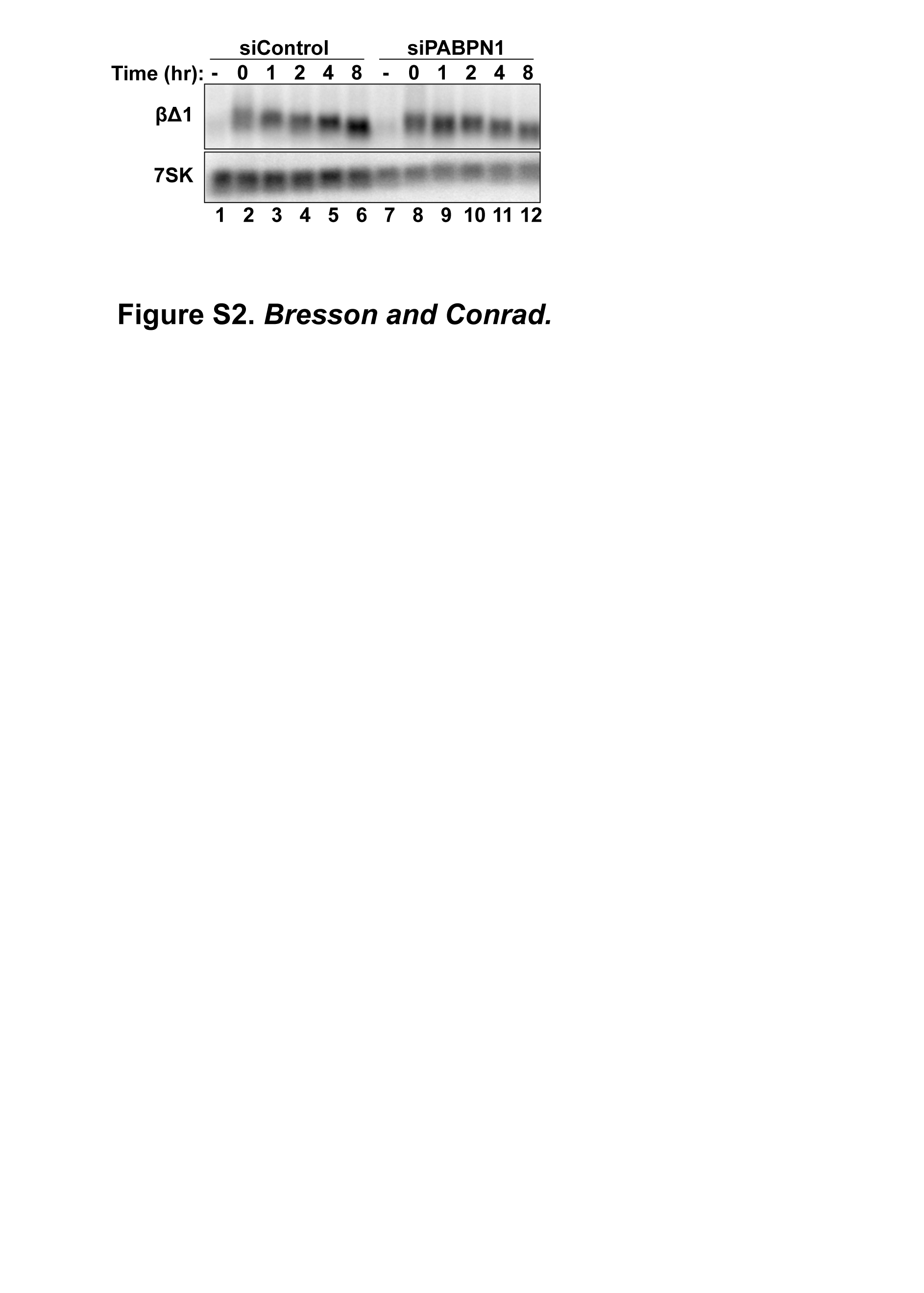

Supplement: Figure S2 — Related to Figure 2. Representative transcription pulse-chase of a spliced (βΔ1) reporter over an eight-hour time course. (TIF) [file pgen.1003893.s002.tif]

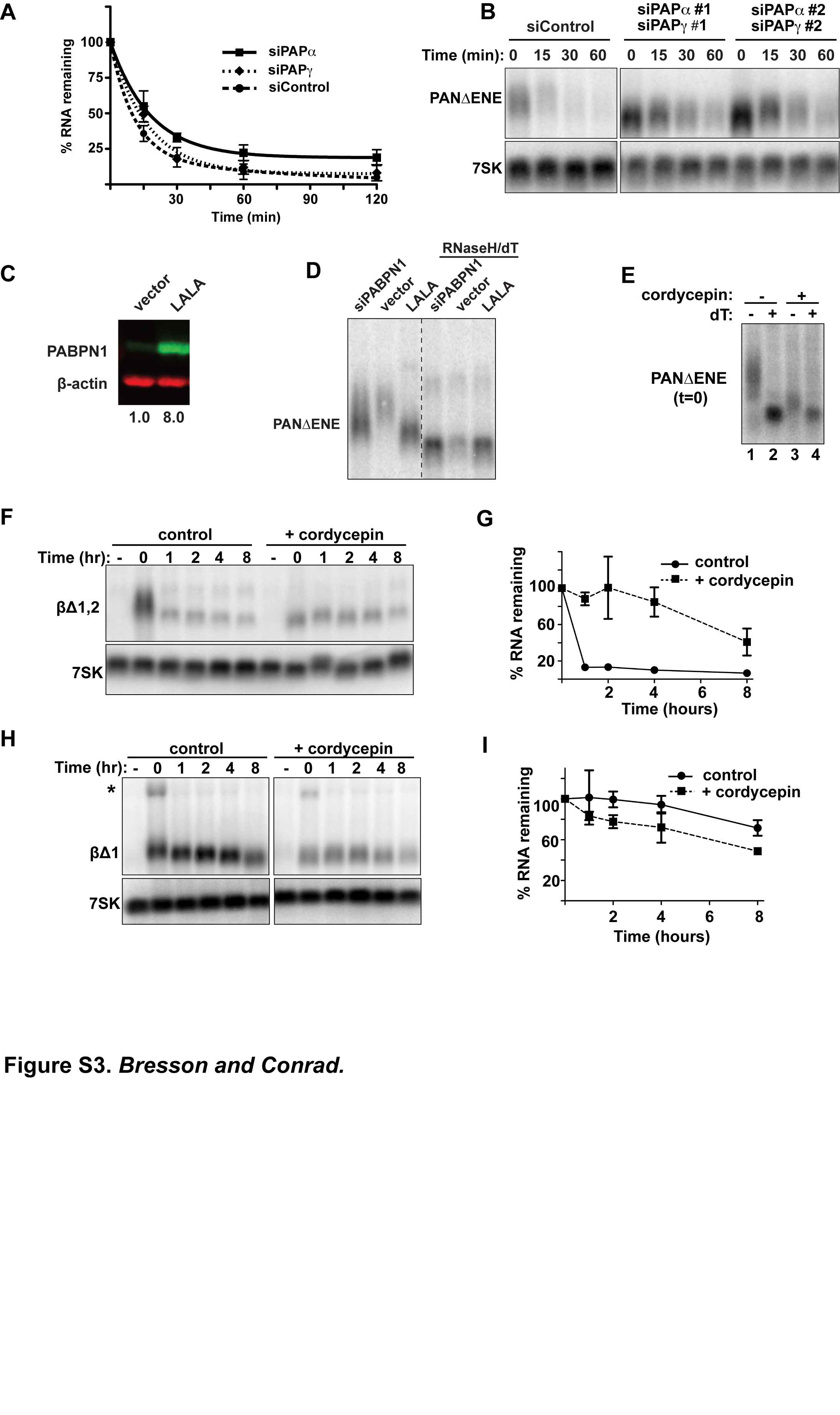

Supplement: Figure S3 — Related to Figure 3. (A) Decay curves of PANΔENE from cells transfected with the indicated siRNAs (n = 3). (B) Northern blot analysis of PANΔENE RNA from cells transfected with independent pools of siRNAs targeting PAPα and PAPγ (C) Quantitative western blot analysis of total lysate from cells transfected with either a vector control, or LALA PABPN1. The blot was probed with antibodies against either PABPN1 (green) or β-actin (red) as a loading control. The relative abundance of PABPN1 normalized to β-actin is indicated below each lane. Importantly, because these values include protein from untransfected cells, they are likely an underestimate of the degree of PABPN1 overexpression. (D) Relative poly(A) tail lengths of PANΔENE RNA following LALA overexpression. RNA from cells treated with siPABPN1 was included for comparison. (E) Relative poly(A) tail lengths of PANΔENE RNA following cordycepin treatment. PANΔENE RNA was cleaved with RNase H targeted by NC30 and oligo(dT) as indicated. The cleaved RNA was analyzed by northern blot using a 3′ end-specific probe. We estimated that the poly(A) tails in the presence of cordycepin were between ∼20–50 nt. (F) Representative transcription pulse assay of βΔ1,2 from cells induced in the presence or absence of cordycepin. (G) Linear interpolation of the results from (C)(n = 3). (H and I) Same as in (F) and (G) except spliced β-globin (βΔ1) was assayed. The same blot and exposure are shown for both panels in (H). (TIF) [file pgen.1003893.s003.tif]

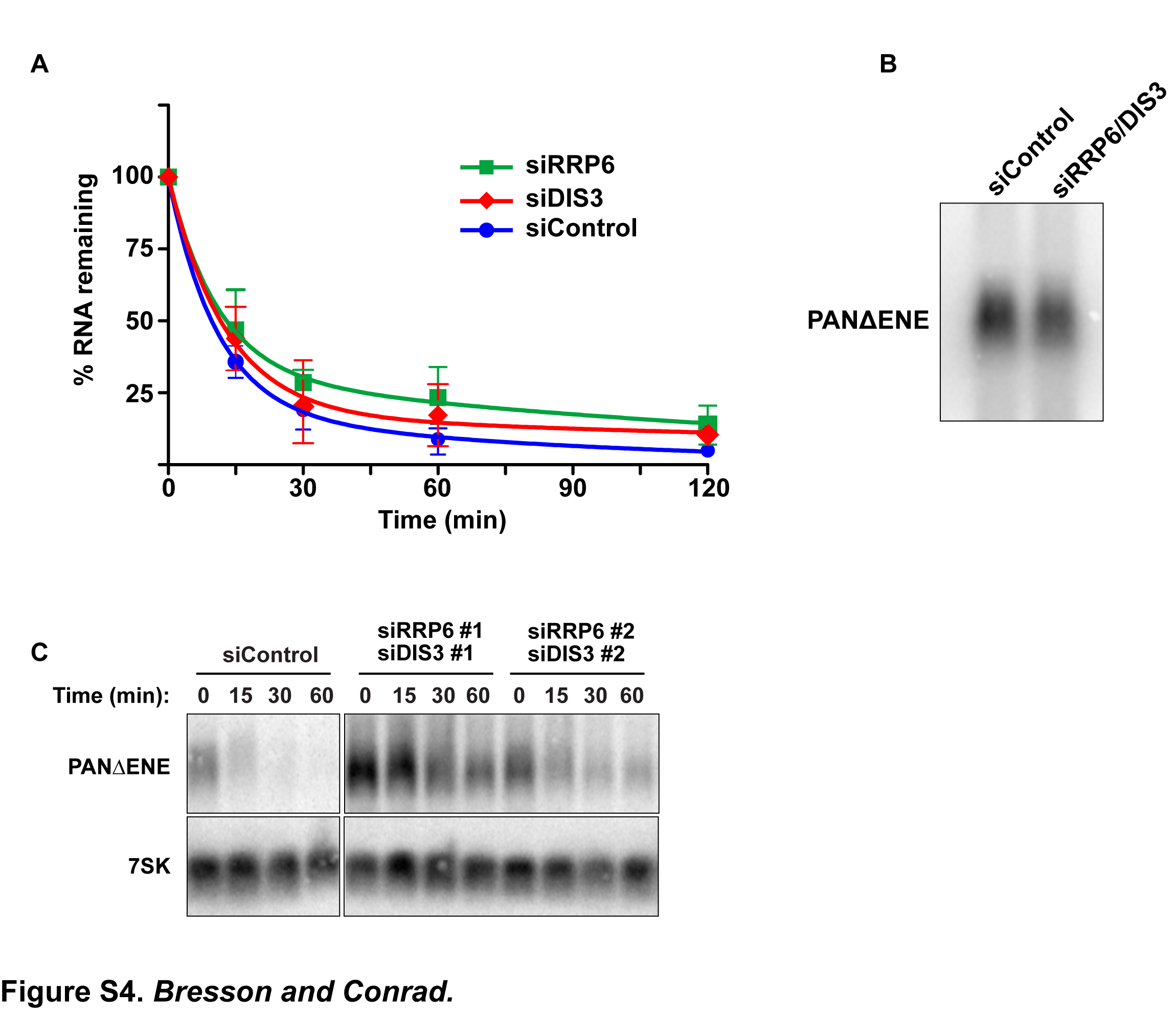

Supplement: Figure S4 — Related to Figure 4. (A) Decay curves of PANΔENE from cells transfected with the indicated siRNAs (n = 3). (B) A comparison of the relative lengths of PANΔENE from cells transfected with the indicated siRNAs. RNA is from the time zero samples (lanes 2 and 10 in Figure 4B). (C) Results from a transcript pulse assay of PANΔENE from cells transfected with two independent pools of siRNAs targeting RRP6 and DIS3. (TIF) [file pgen.1003893.s004.tif]

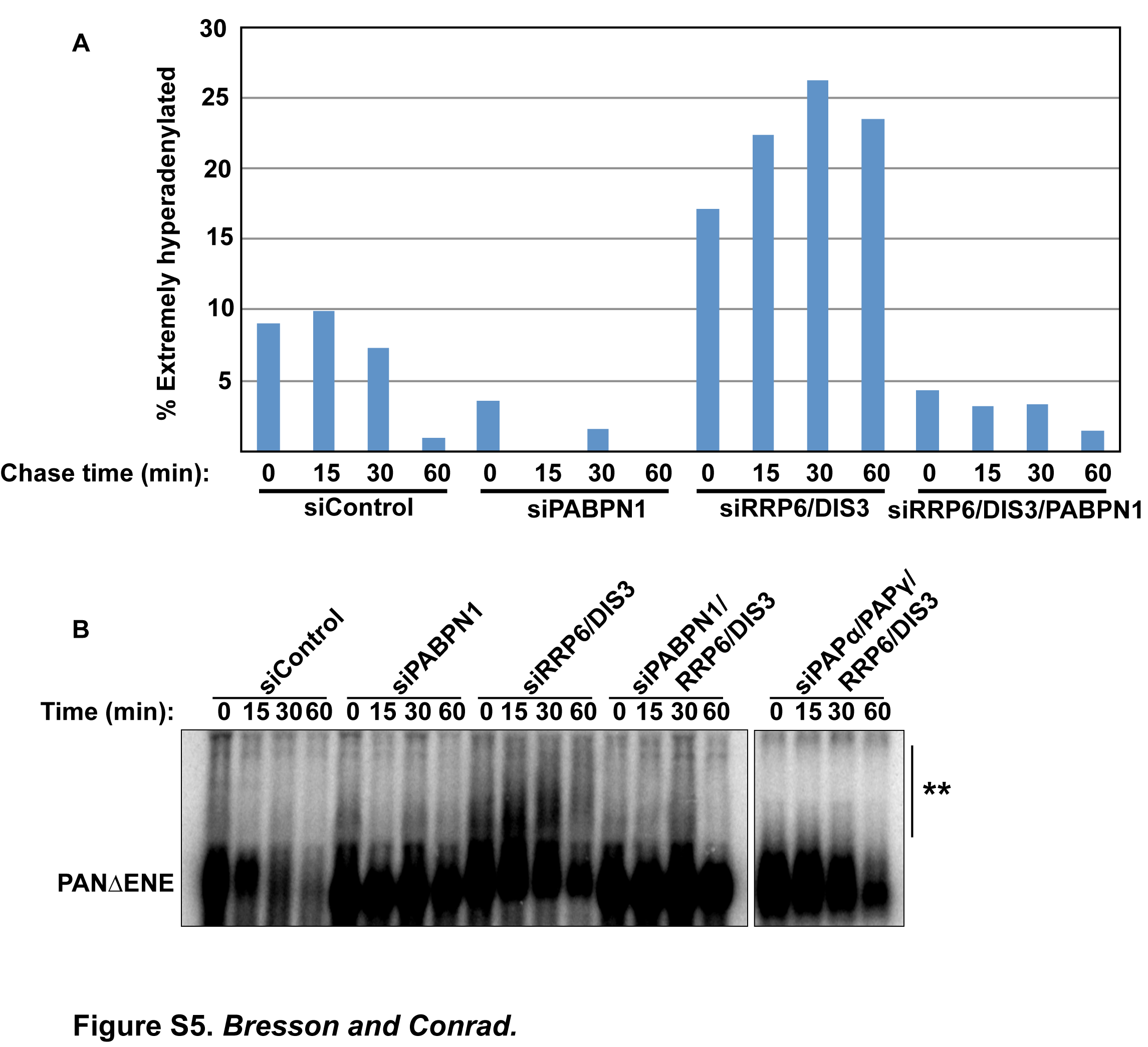

Supplement: Figure S5 — Related to Figure 5. (A) Quantitation of the percent of intronless β-globin which was “extremely hyperadenylated” in each lane of Figure 5D. The “% extremely hyperadenylated” was calculated by boxing the signal from each lane above the primary band, subtracting the background signal, and dividing by the total amount of signal in each lane. (B) Results from a transcription pulse assay of PANΔENE from cells transfected with the indicated siRNAs. Blot was overexposed to reveal the extremely hyperadenylated RNAs (double asterisks). (TIF) [file pgen.1003893.s005.tif]

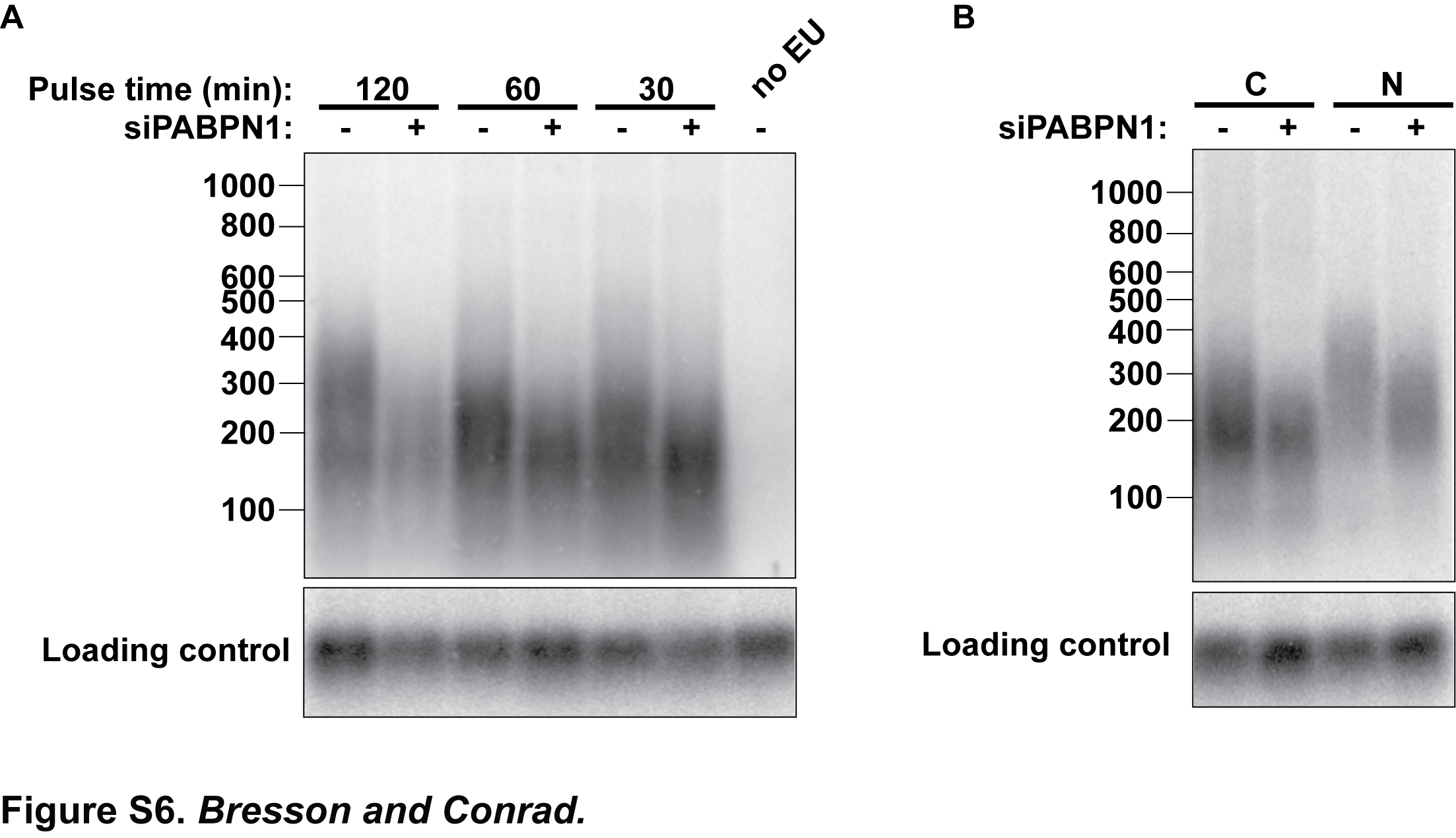

Supplement: Figure S6 — Related to Figure 6. (A) Bulk poly(A) tail analysis from cells transfected with either siControl or siPABPN1. Prior to harvesting the RNA, cells were incubated with EU for the indicated pulse times. To compensate for the shorter pulse times, increasing amounts of total RNA was used for the click reaction: 0.5 µg for the 120′ pulse, 1 µg for the 60′ pulse, and 2 µg for the 30′ pulse and the no EU sample. An exogenously added biotinylated DNA oligo (“Loading control”) was used to control for recovery. Note that this DNA only controls for RNA recovery and loading during the experimental procedure and does not necessarily reflect the total amounts of input RNA. (B) Nuclear/cytoplasmic distribution of poly(A) tails made in the presence or absence of PABPN1. Cells were separated into nuclear and cytoplasmic fractions following a two-hour EU pulse. (TIF) [file pgen.1003893.s006.tif]

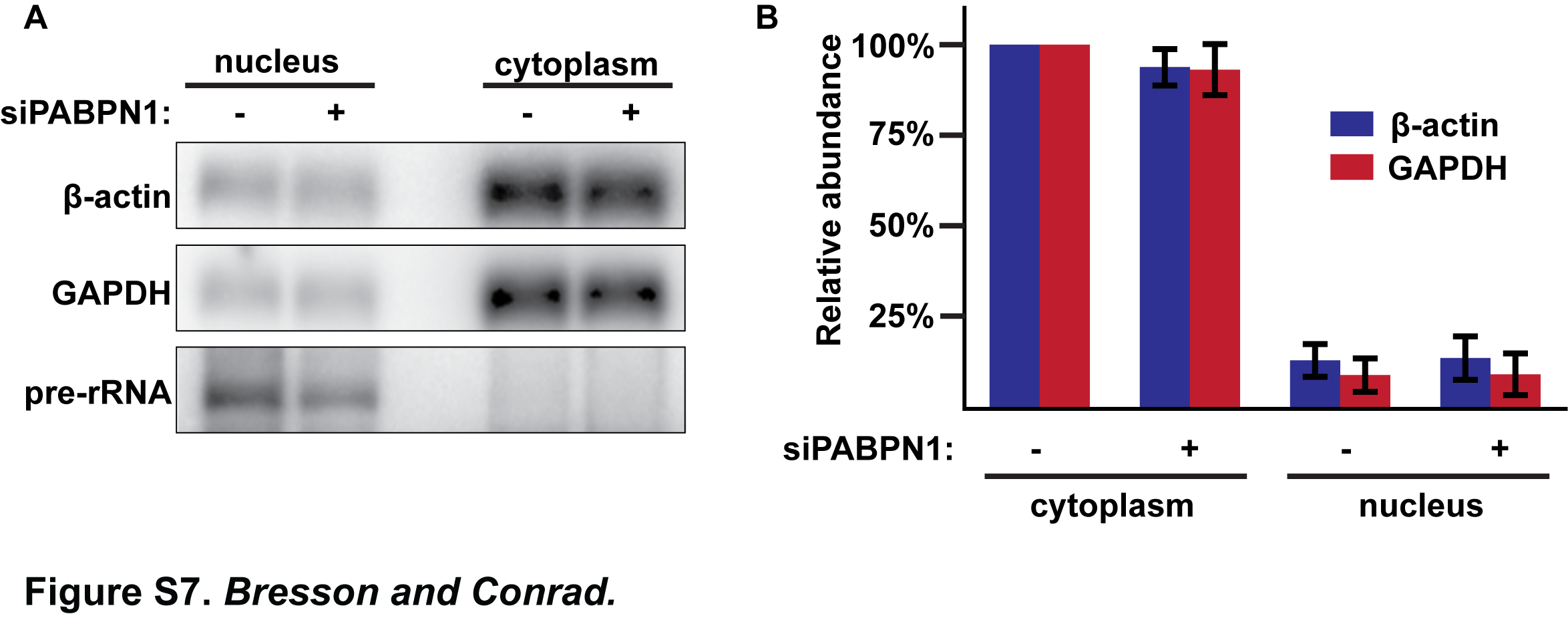

Supplement: Figure S7 — Related to Figure 7. (A) Northern blot analysis of nuclear and cytoplasmic fractions following PABPN1 depletion. The blot was probed for β-actin and GAPDH, as well as pre-ribosomal RNA in order to control for the quality of the fractionation. The amount of RNA loaded was kept constant at a 4∶1 cytoplasmic∶nuclear ratio. (B) Quantification of the results in panel (A) (n = 3). (TIF) [file pgen.1003893.s007.tif]
