# Supplementary material for: The Human Nuclear Poly(A)-Binding Protein Promotes RNA Hyperadenylation and Decay
Source: PLoS Genet. 2013 Oct 17;9(10):e1003893. doi: 10.1371/journal.pgen.1003893 (PMC3798265; doi:10.1371/journal.pgen.1003893)
Supplement: Table S1 — Kinetic parameters estimated from regression analysis of decay data. (DOCX) [file pgen.1003893.s008.docx]

| **Table S1** Kinetic parameters estimated from regression analysis of decay data. | | | | | |
| --- | --- | --- | --- | --- | --- |
| **Reporter RNA** | **siRNA** | **% fast** | **t_1/2_fast (min)** | **t_1/2_slow (min)** | **R^2^** |
| PANΔENE | control* | 94 | 7.4 | >120 | 1.00 |
| PANΔENE | siPABN1 | 6.6 | 15 | 116 | 0.96 |
| βΔ1,2 | control | 73 | 6.4 | 89 | 1.00 |
| βΔ1,2 | siPABN1 | 51 | 5.4 | >120 | 0.98 |
| PANΔENE | control | 84 | 8.4 | 65 | 1.00 |
| PANΔENE | siPAPα/γ | 43 | 15 | 63 | 0.99 |
| PANΔENE | control | 81 | 7.5 | 58 | 1.00 |
| PANΔENE | siDIS3/RRP6 | 38 | 15 | 100 | 0.96 |
| βΔ1,2 | control | 77 | 7 | >120 | 1.00 |
| βΔ1,2 | siPAPα/γ | 63 | 15 | 105 | 1.00 |
| βΔ1,2 | siDIS3/RRP6 | ND | ND | ND | ND |
| **RNA** | **cordycepin** | **% fast** | **t_1/2_fast (min)** | **t_1/2_slow (min)** | **R^2^** |
| PANΔENE | None | 90 | 9.3 | >2hr | 1.00 |
| PANΔENE | T=-2 | 10 | 5.9 | >2hr | 0.97 |
| PANΔENE | T=0 | 67 | 10 | >2hr | 1.00 |
| **RNA** | **Overexpression**  **plasmid** | **% fast** | **t_1/2_fast (min)** | **t_1/2_slow (min)** | **R^2^** |
| PANΔENE | Vector | 88 | 6.6 | >2hr | 1.00 |
| PANΔENE | YAFA | 53 | 15 | >2hr | 0.93 |
| PANΔENE | LALA | ND | ND | ND | ND |
| *siRNA controls were repeated for each new set of experiment. The control is listed in the row above the corresponding targeting siRNA.  ND: not determined | | | | | |
