# Supplementary material for: The Human Nuclear Poly(A)-Binding Protein Promotes RNA Hyperadenylation and Decay
Source: PLoS Genet. 2013 Oct 17;9(10):e1003893. doi: 10.1371/journal.pgen.1003893 (PMC3798265; doi:10.1371/journal.pgen.1003893)
Supplement: Table S2 — siRNAs used in this study. (DOCX) [file pgen.1003893.s009.docx]

| Table S2 siRNAs used in this study | | |
| --- | --- | --- |
| siRNA | Sense sequence | Antisense sequence |
| PABPN1 #1 | GGCCUUAGAUGAGUCCCUAtt | UAGGGACUCAUCUAAGGCCaa |
| PABPN1 #2 | AGUCAACCGUGUUACCAUAtt | UAUGGUAACACGGUUGACUga |
| PAPα #1 | GCCUCGACUUGUCUAUGGAtt | UCCAUAGACAAGUCGAGGCtg |
| PAPα #2 | GUGCUGAUAUUGAUGCGUUtt | AACGCAUCAAUAUCAGCACct |
| PAPγ #1 | CAGCUUAAAUGGUUGUAGAtt | UCUACAACCAUUUAAGCUGcg |
| PAPγ #2 | CCAUAGAUGGGACUCCUAAtt | UUAGGAGUCCCAUCUAUGGat |
| RRP6 #1 | GGAUCGAAGUAAAGUGACUtt | AGUCACUUUACUUCGAUCCtt |
| RRP6 #2 | GAGUAUGAUUUUUACCGAAtt | UUCGGUAAAAAUCAUACUCat |
| DIS3 #1 | GGAGCAUUACUGAAAAGGAtt | UCCUUUUCAGUAAUGCUCCag |
| DIS3 #2 | CAAAAGCAAAGGAAUAGUAtt | UACUAUUCCUUUGCUUUUGaa |
